# Posttranslational Control of PlsB Is Sufficient To Coordinate Membrane Synthesis with Growth in Escherichia coli

**DOI:** 10.1128/mBio.02703-19

**Published:** 2020-08-18

**Authors:** Marek J. Noga, Ferhat Büke, Niels J. F. van den Broek, Nicole C. E. Imholz, Nicole Scherer, Flora Yang, Gregory Bokinsky

**Affiliations:** a Department of Bionanoscience, Kavli Institute of Nanoscience, Delft University of Technology, Delft, The Netherlands; University of Georgia

**Keywords:** fatty acids, lipopolysaccharide, membrane biogenesis, metabolic regulation, phospholipids, posttranslational regulation, ppGpp, proteomics

## Abstract

Every cell must produce enough membrane to contain itself. However, the mechanisms by which the rate of membrane synthesis is coupled with the rate of cell growth remain unresolved. By comparing substrate and enzyme concentrations of the fatty acid and phospholipid synthesis pathways of Escherichia coli across a 3-fold range of carbon-limited growth rates, we show that the rate of membrane phospholipid synthesis during steady-state growth is determined principally through allosteric control of a single enzyme, PlsB. Due to feedback regulation of the fatty acid pathway, PlsB activity also indirectly controls synthesis of lipopolysaccharide, a major component of the outer membrane synthesized from a fatty acid synthesis intermediate. Surprisingly, concentrations of the enzyme that catalyzes the committed step of lipopolysaccharide synthesis (LpxC) do not differ across steady-state growth conditions, suggesting that steady-state lipopolysaccharide synthesis is modulated primarily via indirect control by PlsB. In contrast to steady-state regulation, we found that responses to environmental perturbations are triggered directly via changes in acetyl coenzyme A (acetyl-CoA) concentrations, which enable rapid adaptation. Adaptations are further modulated by ppGpp, which regulates PlsB activity during slow growth and growth arrest. The strong reliance of the membrane synthesis pathway upon posttranslational regulation ensures both the reliability and the responsiveness of membrane synthesis.

## Abstract (precis)

How do bacterial cells grow without breaking their membranes? Although the biochemistry of fatty acid and membrane synthesis is well known, how membrane synthesis is balanced with growth and metabolism has remained unclear. This is partly due to the many control points that have been discovered within the membrane synthesis pathways. By precisely establishing the contributions of individual pathway enzymes, our results simplify the model of membrane biogenesis in the model bacterial species Escherichia coli. Specifically, we found that allosteric control of a single enzyme, PlsB, is sufficient to balance growth with membrane synthesis and to ensure that growing E. coli cells produce sufficient membrane. Identifying the signals that activate and deactivate PlsB will resolve the issue of how membrane synthesis is synchronized with growth.

## INTRODUCTION

All cells build and expand their membranes at a pace that must be coordinated with growth of the cell, as excessive or insufficient membrane production can be fatal. In Gram-negative bacteria, construction of the double membrane from fatty acid precursors demands coordination between phospholipid (PL) and lipopolysaccharide (LPS) synthesis pathways (1, 2) as well as with protein synthesis, which supplies the lipoproteins that tether the outer membrane to the peptidoglycan cell wall (3). Many elements of membrane synthesis regulation that act on either transcription or enzymes activities have been proposed or identified. However, how each of these individual elements is used to regulate membrane synthesis during steady-state growth remains unclear (4).

The issue of how membrane construction is coordinated with growth can be approached by considering how the metabolic pathways that synthesize membrane building blocks (PL and LPS) are regulated. Biosynthetic fluxes are regulated either by control of enzyme concentrations or by direct control of enzyme activity. Examples of both forms of regulation can be readily found: in Escherichia coli, the steady-state protein synthesis rate is controlled by ribosome concentration, which is transcriptionally regulated to balance amino acid supply with translation demand (5). In contrast, fluxes through central carbon metabolism are controlled by concentrations of substrates and inhibitors (i.e., posttranslational control) rather than by changes in enzyme concentration (6, 7). Posttranslational control is also known to contribute to membrane synthesis regulation (8–13); however, how cells use transcriptional and posttranslational control to coordinate membrane synthesis with growth has never been defined well.

In order to understand how the model Gram-negative species Escherichia coli coordinates membrane synthesis with growth, we quantified substrates and enzymes of the fatty acid and PL synthesis pathways under both steady-state and dynamic conditions. We found that posttranslational control of the first enzyme in the PL synthesis pathway, PlsB, is sufficient to ensure steady-state regulation of PL synthesis. In contrast, transcriptional regulation maintains stable enzyme concentrations regardless of growth rate. Furthermore, due to feedback regulation of the fatty acid pathway, PlsB exerts strong control over LPS synthesis via its effects on concentrations of an LPS fatty acid precursor.

## RESULTS

### The PL to biomass ratio varies inversely with μ.

**FIG 1:**
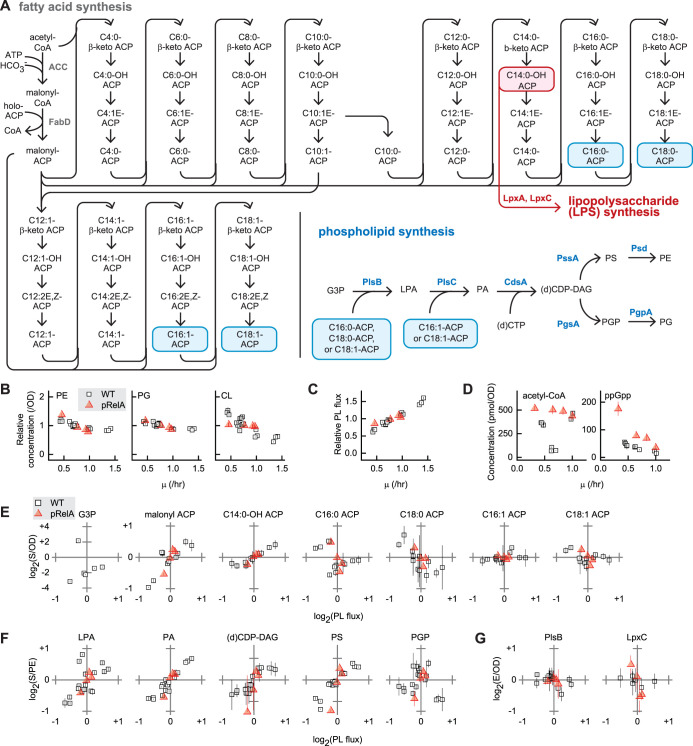
Characterization of the E. coli fatty acid and PL synthesis pathways during steady-state growth. (A) The fatty acid and PL synthesis pathways. Long-chain fatty acids are transferred from C16:0-ACP, C18:0-ACP, or C18:1-ACP (highlighted in blue) to *sn-*glycerol-3-phosphate (G3P) by PlsB to yield lysophosphatidic acid (LPA), which is acylated with C16:1-ACP or C18:1-ACP to generate phosphatidic acid (PA). PA is converted to (d)CDP-diacylglycerol [(d)CDP-DAG] with either CTP or dCTP. At the branch point in PL synthesis, (d)CDP-DAG is converted to phosphatidylglycerol phosphate (PGP), which is dephosphorylated to yield PG (∼25% of total PL). (d)CDP-DAG is also a precursor of phosphatidylserine (PS), which is decarboxylated to yield PE (∼70% of total PL). PG is further converted to CL (∼5% of total PL). C14:0-OH-ACP (highlighted in red) is a precursor for the synthesis of lipopolysaccharide (LPS), the major component of the outer leaflet of the outer membrane. (B and C) Growth rate (μ)-dependent abundance of PG, PE, and CL per OD (B) and μ dependence of PL flux (C), normalized to average values for each strain. (D) Steady-state concentrations of acetyl-CoA and ppGpp. (E, F, and G) Correlations of G3P and ACP concentrations (E), PL intermediate concentrations (F), and PlsB and LpxC concentrations (G) with PL flux. Concentrations of soluble substrates and enzymes were calculated using cell volume (proportional to OD), while PL intermediate concentrations were calculated using membrane volume, which is proportional to total PE. All concentrations and fluxes were normalized and log(2) transformed for comparison. All data points and error bars represent averages and standard deviations, respectively, of measurements of three samples from one culture. For wild-type (WT) PL and PL intermediates, three independent biological replicates of each condition are depicted; for ACP species and PlsB, two independent biological replicates are depicted; for nucleotides, LpxC, and G3P, data from one experiment per condition are shown. For pRelA, one measurement per inducer concentration is shown.

Lipid precursors of both LPS and PL are synthesized in the cytosol as fatty acyl thioesters covalently attached to acyl carrier protein (ACP). Fatty acid synthesis is initiated by carboxylation of acetyl coenzyme A (acetyl-CoA) by the acetyl-CoA carboxylase complex (ACC) to produce malonyl-CoA, from which the malonyl group is transferred to holo-ACP by the activity of FabD. Malonyl-ACP is used to elongate fatty acid through iterated cycles, beginning with condensation with acyl-ACP, followed by reduction, dehydration, and reduction. The first step in PL synthesis is catalyzed by the membrane-bound enzyme PlsB, which synthesizes lysophosphatidic acid (LPA) from long-chain ACP and *sn*-glycerol-3 phosphate (G3P). G3P is produced either from glyceraldehyde-3-phosphate, an intermediate of central carbon metabolism, or via glycerol catabolism (Fig. 1A). We grew cultures of E. coli NCM3722 in 6 defined media that support a 3-fold range of growth rates (μ). Quantities of major PL species phosphatidylglycerol (PG), phosphatidylethanolamine (PE), and cardiolipin (CL) per unit biomass (as determined by culture optical density [OD] [14]) decreased slightly with increasing μ (Fig. 1B), consistent with previous observations in E. coli and other bacteria (15, 16). The PG/PE ratio remained constant across all μ. We quantified steady-state PL flux by multiplying total PE liquid chromatography/mass spectrometry (LC/MS) counts by μ, which closely approximates the total PL synthesis rate as PE turnover is slow relative to synthesis (17) and the PG/PE ratio is stable. PL flux increases by 2-fold as μ increases by 3-fold (Fig. 1C). The higher PL-to-biomass ratio of slow-growing cells likely reflects their higher surface-to-volume ratio (18).

### Growth regulates PL flux via posttranslational control of PlsB.

We first sought to identify the control points of PL synthesis by determining correlations between PL flux and concentrations of pathway intermediates during steady-state growth. If PL and fatty acid synthesis rates are controlled by substrate concentrations, a positive correlation between substrate concentrations and flux should be observed. We quantified nucleotide, G3P, ACP, and PL synthesis intermediates using LC/MS (19). No correlation was observed between concentrations of the fatty acid precursor acetyl-CoA and μ (Fig. 1D). We also found no significant positive correlation between PL flux and PlsB substrate C16:0-ACP or C18:1-ACP (Pearson correlation coefficient *r* = <0.4, two-tailed test of significance *P* = >0.1), while C18:0-ACP concentrations correlated negatively with PL flux (*r* = −0.76, *P* < 0.01) (Fig. 1E; see also Table 1). Although G3P concentrations were found to correlate with PL flux (*r* = >0.99, *P* = <0.01), growth in glycerol medium increased G3P by 20-fold without increasing PL abundance or flux. The absence of any significant positive correlation between ACP substrate concentrations and PL flux indicates that steady-state PL synthesis is not controlled by substrate concentrations.

**TABLE 1:** Pearson correlation coefficients and significance determined for PL flux and species abundance during steady-state growth in wild-type E. coli^*a*^

Substrate or enzyme	*n*	Pearson correlation coefficient (*r*)	Significance (*P*, two-tailed)
Substrate			
Holo-ACP	12	−0.76	<0.01
Malonyl-ACP	12	0.80	<0.01
G3P	5	0.99	<0.01
C14:0-OH-ACP	12	0.94	<10^-4^
C14:0-ACP	12	−0.16	0.6
C16:0-ACP	12	−0.42	0.2
C18:0-ACP	12	−0.76	<0.01
C16:1-ACP	12	0.42	0.2
C18:1-ACP	12	−0.38	0.2
LPA	18	0.68	<0.01
PA	18	0.95	<10^−4^
(d)CDP-DAG	18	0.89	<10^−4^
PS	18	0.91	<10^−4^
PGP	15	0.61	0.015

Enzyme			
LpxC	6	−0.4	0.4
PlsB	12	−0.4	0.2
PlsC	12	0.63	0.03
CdsA	12	0.90	<10^−4^
PssA	12	−0.4	0.2
Psd	12	0.2	0.6
PgsA	12	−0.7	0.01
PgpA	12	0.9	<10^−4^
AccA	12	0.70	0.01
AccB	12	0.38	0.2
AccC	12	0.50	0.1
AccD	12	0.21	0.5
AcpP	12	0.04	0.9
FabA	12	0.29	0.4
FabB	12	0.28	0.4
FabD	12	0.35	0.3
FabF	12	0.76	<0.01
FabG	12	0.68	0.02
FabH	6	0.49	0.3
FabI	12	0.82	<0.001
FabZ	12	0.77	<0.01
GpsA	12	−0.69	0.01

a *n* indicates the number of independent measurements used to calculate the correlation coefficients. Shaded cells indicate *P* values of <0.05. G3P data were calculated with measurement in glycerol medium excluded. PGP data were calculated with measurements in glucose plus cas-amino acids excluded.

In contrast to PL precursors, concentrations of the fatty acid initiation and elongation substrate malonyl-ACP correlated with PL flux (Fig. 1E; *r* *= *0.80, *P* = <0.01) (Table 1), while the concentrations of most saturated ACP species remained relatively constant (see Fig. S1 in the supplemental material), suggesting that the fatty acid initiation and elongation reactions are controlled primarily by malonyl-ACP concentrations and thus by the rate of malonyl-ACP synthesis by ACC and FabD. Concentrations of C14:0-OH-ACP, the lipid precursor of LPS, also correlated with PL flux (*r *= 0.94, *P* = <10^−4^), suggesting that PL synthesis is naturally coupled with LPS synthesis via concentrations of C14:0-OH-ACP (Fig. 1E). Concentrations of holo-ACP, the most abundant ACP species, declined with increasing PL flux (*r* = −0.76, *P* = <0.01) (Table 1), as levels of fatty acid synthesis intermediates increased due to increased fatty acid flux.

10.1128/mBio.02703-19.2 FIG S1 Steady-state concentrations of ACP of E. coli NCM3722 in six media and of E. coli pRelA* in glucose cultures with titrated RelA* expression (triangles). Concentrations and PL fluxes are normalized to average across each individual replicate series. Error bars represent standard deviations of results from 3 sampling replicates. Two series of independent biological replicates were obtained for E. coli NCM3722. Download FIG S1, PDF file, 0.2 MB. Copyright © 2020 Noga et al. 2020 Noga et al. This content is distributed under the terms of the Creative Commons Attribution 4.0 International license.

As with substrates, positive correlations between enzyme concentrations and reaction flux may indicate that a reaction rate is controlled by enzyme concentrations, i.e., via transcriptional control. We measured concentrations of fatty acid synthesis pathway enzymes using LC/MS to determine whether fatty acid flux is coupled with μ by transcription-based regulation. While concentrations of one of the four ACC subunits (AccA) and several of the fatty acid synthesis pathway enzymes correlated with PL flux (Table 1; see also Fig. S2), the increases were small (30%) relative to the PL flux increase (2-fold). We infer that the rate of malonyl-ACP synthesis and thus the rate of fatty acid synthesis are determined primarily by posttranslational control of ACC. The negative correlation between C18:0-ACP and PL flux is consistent with long-chain ACP inhibiting malonyl-CoA synthesis by ACC via negative-feedback inhibition (20).

10.1128/mBio.02703-19.3 FIG S2 Steady-state concentrations of fatty acid and PL synthesis enzymes of E. coli NCM3722 in six media and of E. coli pRelA* in glucose cultures with titrated RelA* expression (triangles). Concentrations and PL fluxes are normalized to average across each individual replicate series. Error bars represent standard deviations of results from 3 sampling replicates. Two series of independent biological replicates were obtained for E. coli NCM3722. Download FIG S2, PDF file, 0.4 MB. Copyright © 2020 Noga et al. 2020 Noga et al. This content is distributed under the terms of the Creative Commons Attribution 4.0 International license.

Unlike long-chain ACP, the concentrations of all PL synthesis intermediates downstream of PlsB were found to correlate positively with PL flux (Fig. 1F; *r *= 0.61 to 0.99) (Table 1; see also Fig. S3). The close match between the increase in the concentration and the level of PL flux is consistent with the rates of these reactions being under substrate control. The contrast between the trends in concentrations of the substrates and the products of PlsB implies that PlsB activity alone controls PL synthesis. PlsB activity might be regulated either via transcriptional control of PlsB concentration or via posttranslational control of PlsB activity. Transcription of *plsB* is controlled by the membrane stress-activated sigma factor RpoE (21) and by the μ-sensitive regulator guanosine tetraphosphate (ppGpp) (22). Concentrations of ppGpp are inversely correlated with μ (Fig. 1D), implying that PL flux may be coupled to μ by transcriptional control of the *plsB* gene by ppGpp. However, we found no correlation between PL flux and PlsB concentration (Table 1, *P* = >0.1). Instead, PlsB concentrations were nearly constant across all conditions studied (Fig. 1G). The invariance of PlsB concentrations over a 2-fold range of PL flux indicates that PlsB activity is regulated mainly via posttranslational control. Unexpectedly, concentrations of LpxC, which catalyzes the committed step in the LPS pathway (23), also did not correlate with PL flux (Table 1, *P* = >0.1) and also remained stable (Fig. 1G), indicating that changes in LPS flux between steady-state growth conditions are not driven by changes in LpxC concentrations.

10.1128/mBio.02703-19.4 FIG S3 Steady-state concentrations of PL synthesis intermediates of E. coli NCM3722 in six media and of E. coli pRelA* in glucose cultures with titrated RelA* expression (triangles). Concentrations and PL fluxes are normalized to average across each individual replicate series. Error bars represent standard deviations of results from 3 sampling replicates. Three series of independent biological replicates were obtained for E. coli NCM3722. Download FIG S3, PDF file, 0.2 MB. Copyright © 2020 Noga et al. 2020 Noga et al. This content is distributed under the terms of the Creative Commons Attribution 4.0 International license.

μ can be modulated independently of nutrient conditions by artificially altering levels of ppGpp. Titrating ppGpp above basal concentrations, but below the concentrations that occur during starvation, reduces steady-state μ by reducing rRNA synthesis (24). Furthermore, PlsB activity is inhibited at the high ppGpp concentrations observed during amino acid starvation (25). We titrated μ by expressing the catalytic domain of the ppGpp synthesis enzyme RelA (RelA*) from an inducible promoter (P_Tet_) in glucose medium. As previously observed (24), RelA*-titrated cultures exhibited ppGpp concentrations that were elevated 2-fold above those seen with wild-type cultures growing at similar rates (Fig. 1D). Trends in PL abundance, PL flux, ACP species, PL intermediates, and PL synthesis enzyme concentrations in ppGpp-limited cultures closely followed the trends observed when μ was adjusted by carbon source (Fig. 1B, C, and E to G) (see also Fig. S1, S2, and S3). Notably, despite ppGpp regulation of *plsB* transcription, concentrations of PlsB also did not substantially vary with increasing concentrations of steady-state ppGpp. Interestingly, LpxC concentrations decreased with increasing PL flux under these conditions (Fig. 1G). The trends in substrate, enzyme, and intermediate concentrations observed in ppGpp-limited cultures are consistent with growth regulating PL flux via posttranslational control—not transcriptional control—of PlsB.

### Mathematical modeling supports PlsB control of steady-state PL and LPS synthesis.

**FIG 2:**
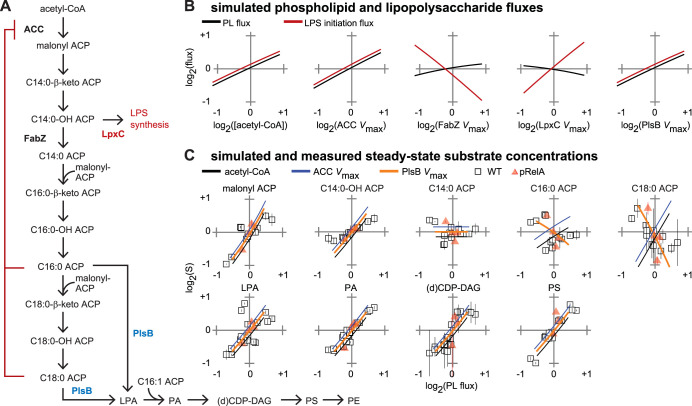
Simulated PL synthesis and experimental data identify PlsB as a site of PL synthesis control during steady-state growth. (A) Reactions simulated in the model. For simplicity, only reactions occurring late in the saturated fatty acid pathway are included, and branching of the PL pathway into PE and PG is not included. Each reaction is modeled as an irreversible one- or two-substrate Michaelis-Menten reaction. ACC is competitively inhibited by C16:0-ACP and C18:0-ACP. Reactions catalyzed by FabI/FabZ and LpxA/LpxC are considered in the model as single reactions (represented in the diagram by “FabZ” and “LpxC,” respectively). (B) Response of PE and LPS fluxes to varying *V*_max_ of pathway enzymes and acetyl-CoA concentrations. Variations (4-fold) in *V*_max_ of all other reactions tested did not change PE or LPS flux (not shown). (C) Simulated changes in metabolite concentrations in response to variations in PlsB and ACC *V*_max_ and acetyl-CoA (line plots) overlaid on experimentally measured concentrations (scatterplots; data are from Fig. 1). Line plots are offset to prevent overlap. Differential equations and parameters of the simulation are provided in Text S1 and Table S1.

To test whether regulation of PlsB activity is sufficient to control steady-state PL synthesis, we constructed a simplified differential equation model that describes fatty acid, LPS initiation, and PL biosynthesis (Fig. 2A; see also Text S1 and Table S1). The model includes competitive inhibition of ACC by both C16:0-ACP and C18:0-ACP as the sole regulatory interactions. The model also includes a branch point at C14:0-OH-ACP into LPS synthesis. LPS initiation is represented in the model by a single step that combines reactions catalyzed by LpxA and LpxC, as LpxC catalyzes the first irreversible reaction in the LPS pathway. Concentrations of G3P and C16:1-ACP are fixed in the model to reflect experimental observations of PlsB saturation by G3P and invariance of C16:1-ACP concentrations, respectively (see Text S1 in the supplemental material for model details, parameter sets, and sensitivity analyses).

10.1128/mBio.02703-19.1 TEXT S1 Description of the steady-state differential equation model depicted in Fig. 2. Download Text S1, DOCX file, 0.02 MB. Copyright © 2020 Noga et al. 2020 Noga et al. This content is distributed under the terms of the Creative Commons Attribution 4.0 International license.

10.1128/mBio.02703-19.10 TABLE S1 Differential equations used to define steady-state fluxes in the model. Note that ppGpp inhibition of PlsB was not considered in the steady-state calculations (i.e., the ppGpp concentration was set to 0 μM). Download Table S1, PDF file, 0.1 MB. Copyright © 2020 Noga et al. 2020 Noga et al. This content is distributed under the terms of the Creative Commons Attribution 4.0 International license.

We used the model to identify enzymes and substrates that exert control over PL and LPS synthesis. Increasing either ACC or PlsB *V*_max_ by 4-fold increased simulated PL and LPS fluxes in parallel by 2-fold (Fig. 2B), while changing *V*_max_ of all other enzymes in the simulated pathway had little or no effect on simulated PL flux. The insensitivity of PL flux to *V*_max_ of all fatty acid and PL synthesis enzymes aside from ACC and PlsB suggests that the experimentally observed positive correlations between enzyme concentrations and PL flux did not actually result in increased PL flux. The unexpectedly strong control of LPS flux by PlsB is due to the natural coupling between PL flux and concentrations of the LPS synthesis substrate C14:0-OH-ACP. Changes in C14:0-OH-ACP dehydration/reduction *V*_max_ (catalyzed in E. coli by FabZ and FabI, respectively) and LPS initiation *V*_max_ (catalyzed by LpxA and LpxC) exert strong and opposing forms of control over LPS flux. However, LpxC and FabZ do not exert significant control over PL synthesis, indicating that substantial flux can be diverted into the LPS pathway without depleting that required for PL synthesis. Of the two substrates considered in the model (acetyl-CoA and C16:1-ACP), only acetyl-CoA affects PL and LPS flux (Fig. 2B).

To determine which of the two enzymes with nonnegligible levels of PL flux control—ACC or PlsB—actually controls PL flux during steady-state growth, we compared the predicted trends in substrate concentrations caused by simulated *V*_max_ variations against experimentally observed trends. The predicted trends driven by ACC and PlsB *V*_max_ variation closely reproduce most experimentally observed trends in both fatty acid and PL intermediate concentrations (Fig. 2C). However, variations in ACC and PlsB *V*_max_ cause opposing trends in predicted concentrations of PlsB substrates C16:0-ACP and C18:0-ACP, allowing ACC regulation to be distinguished from PlsB-based regulation. Increasing ACC *V*_max_ causes C16:0-ACP and C18:0-ACP concentrations to increase with PL flux, which contradicts our experimental observations. However, increasing PlsB *V*_max_ is predicted to decrease C16:0-ACP and C18:0-ACP concentrations, a trend that better follows the experimentally observed trends (Fig. 2C). We therefore conclude that PL synthesis is primarily regulated by PlsB during steady-state growth. Our model also suggests that PlsB control over steady-state LPS flux (achieved by its tight control over C14:0-OH-ACP concentrations) may be sufficient to couple LPS synthesis with growth as well.

### Translation inhibition causes carbon overflow into fatty acid synthesis.

We set out to evaluate whether a known regulator of PL synthesis, ppGpp, is able to directly regulate PlsB activity during steady-state growth. High concentrations of ppGpp lead to PlsB inhibition *in vivo* (25), although it remains unclear whether this inhibition acts via a ppGpp-PlsB interaction or via an indirect route. Low concentrations of ppGpp correlated inversely with μ (Fig. 1D). The notion that ppGpp might directly control PL synthesis even at the low basal concentrations present during steady-state growth was proposed previously but never tested (26). Levels of acyl-ACP substrates of PlsB increased as ppGpp was titrated upwards with RelA* whereas the level of the product of PlsB (LPA) decreased, consistent with ppGpp inhibiting flux into the PL pathway (Fig. 1E and F) and closely following simulated variations in PlsB *V*_max_ (Fig. 2C). Therefore, changes in μ driven by substringent concentrations of ppGpp must somehow influence steady-state PlsB activity. However, the mode of control by ppGpp (transcriptional control of *plsB*, posttranslational inhibition of PlsB, or indirect control via an unknown regulator of PlsB) cannot be determined from steady-state data alone.

**FIG 3:**
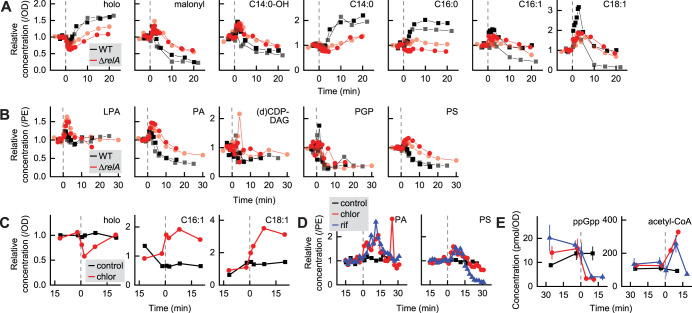
Responses of the fatty acid and PL synthesis pathways to translation inhibition. (A and B) Responses of ACP intermediates (A) and PL intermediates (B) to mupirocin. Mupirocin was added at 0 min (indicated by dashed lines) to glucose cultures of E. coli wild-type and Δ*relA* NCM3722. Each point represents one measurement from a time series collected from an independent culture. Two biological replicate series for each strain are depicted. (C and D) Addition of translation inhibitor chloramphenicol or the transcription inhibitor rifampin to glycerol cultures of wild-type E. coli (indicated by dashed lines at 0 min) causes an influx of carbon into the fatty acid pathway, as suggested by a decrease in holo-ACP levels and an increase in the levels of unsaturated long-chain ACP species (C). The pulse of carbon continues into the PL synthesis pathway, as indicated by transient increases in total PA and PS levels (D). Each trajectory indicated in panels C and D was obtained from a single culture. (E) Both chloramphenicol and rifampin trigger a rapid decrease in ppGpp levels and accumulation of ACC and FabH substrate acetyl-CoA. Points and error bars in panel E represent averages and standard deviations of results from triplicate samples, respectively, from individual experiments.

We first wished to observe the effects of high concentrations of ppGpp on the fatty acid and PL synthesis pathways. Synthesis of high concentrations of ppGpp by RelA (the stringent response) is triggered by any stress or starvation conditions that lead to the specific biochemical cue of uncharged tRNA bound to the ribosome. During the stringent response, ppGpp accumulates by more than 10-fold over basal concentrations and PL synthesis rates are reduced by approximately half (25, 26), likely due to PlsB inhibition (27). We triggered ppGpp synthesis by adding the tRNA aminoacylation inhibitor mupirocin to glucose cultures of wild-type E. coli. To distinguish effects of ppGpp from effects of translation inhibition, mupirocin was also added to a culture of Δ*relA* E. coli. Mupirocin caused a 10-fold accumulation of ppGpp in the wild-type strain within 1 min to over 400 pmol/OD, reaching >800 pmol/OD after 3 min (Fig. S4). Unexpectedly, mupirocin treatment also transiently increased concentrations of malonyl-ACP and all hydroxyl-ACP species at the expense of holo-ACP in both strains (Fig. 3A; see also Fig. S5). The increases in malonyl-ACP and hydroxyl-ACP concentrations were matched by a corresponding increase in PL synthesis intermediates, suggesting that mupirocin had diverted a pulse of carbon into the fatty acid and PL synthesis pathways (Fig. 3B; see also Fig. S6). In the wild-type strain, the pulse of fatty acid synthesis activity was rapidly followed by C14:0-ACP, C16:0-ACP, and C18:0-ACP accumulation, consistent with ppGpp inhibition of PlsB (Fig. 3A; see also Fig. S5). C16:0-ACP accumulation was followed in turn by an increase in holo-ACP and a decrease in malonyl-ACP levels, consistent with ACC inhibition (Fig. 3A). PL intermediates phosphatidic acid (PA), PS, and PGP were rapidly depleted in the wild-type strain after briefly increasing in abundance (Fig. 3B). The transient carbon influx briefly shifts the population of the acyl chains incorporated into PL toward longer-chain fatty acids, likely due to increased malonyl-ACP concentrations favoring fatty acid elongation over membrane incorporation by PlsB and PlsC (Fig. S6). While suppression of fatty acid synthesis in the wild-type strain can be attributed to ppGpp, it is unclear what had attenuated the carbon influx in the Δ*relA* strain; however, C14:0-ACP accumulation after 10 min likely contributed to ACC inhibition. Inhibition of fatty acid elongation also depleted the LPS precursor C14:0-OH-ACP, which is expected to decrease LPS synthesis in parallel with PL synthesis (Fig. 3A).

10.1128/mBio.02703-19.5 FIG S4 (A) Dynamics of nucleotide pools of wild-type E. coli after mupirocin addition (dashed line at *t* = 0 minutes). Data points indicate individual measurements during a single time series. (B) Response of nucleotide pools of wild-type and Δ*relA* strains to mupirocin, added at *t* = 0. Error bars represent standard deviations of results from 3 sampling replicates from individual time series. Download FIG S4, PDF file, 0.4 MB. Copyright © 2020 Noga et al. 2020 Noga et al. This content is distributed under the terms of the Creative Commons Attribution 4.0 International license.

10.1128/mBio.02703-19.6 FIG S5 Response of ACP species (counts per OD unit) to mupirocin at *t* = 0 in wild-type and Δ*relA* E. coli. Values are normalized such that the concentrations before *t* = 0 are averaged to a value of 1. Data points indicate individual measurements from a single time series. Two independent biological replicates per strain are shown. Download FIG S5, PDF file, 0.7 MB. Copyright © 2020 Noga et al. 2020 Noga et al. This content is distributed under the terms of the Creative Commons Attribution 4.0 International license.

10.1128/mBio.02703-19.7 FIG S6 Response of individual PL species (counts per total PE) to mupirocin at *t* = 0 in wild-type and Δ*relA* E. coli. Values are normalized such that the concentrations before *t* = 0 are averaged to a value of 1. Data points indicate individual measurements from a single time series. Two independent biological replicates per strain are shown. Download FIG S6, PDF file, 0.6 MB. Copyright © 2020 Noga et al. 2020 Noga et al. This content is distributed under the terms of the Creative Commons Attribution 4.0 International license.

As malonyl-ACP levels increased transiently after mupirocin addition in both the wild-type and Δ*relA* strains, we hypothesized that translation inhibition somehow diverts carbon into lipid synthesis. We added the ribosome inhibitor chloramphenicol and the transcription initiation inhibitor rifampin to glycerol cultures of wild-type E. coli. Both compounds inhibit translation via mechanisms that suppress ppGpp synthesis. As with mupirocin treatment of the Δ*relA* strain, chloramphenicol triggered a rapid decrease in holo-ACP and an increase in long-chain unsaturated ACP species C16:1-ACP and C18:1-ACP levels (Fig. 3C). Both antibiotics triggered an increase in the levels of PL synthesis intermediates PA and PS that resembled the response of the Δ*relA* strain to mupirocin (Fig. 3D).

What might have caused the transient increase in fatty acid synthesis observed after translation inhibition? Interestingly, addition of both rifampin and chloramphenicol increased acetyl-CoA concentrations in glycerol cultures by 3-fold (Fig. 3E), suggesting a possible cause. Acetyl-CoA concentrations also increased in both wild-type and Δ*relA* strains after mupirocin treatment in glucose medium, before decreasing ∼30% in the wild-type strain (Fig. S4). While it is unclear why translation inhibition would increase acetyl-CoA concentrations, the observed response of the fatty acid pathway is consistent with our mathematical model, which predicts fatty acid flux to be highly sensitive to changes in acetyl-CoA concentrations (Fig. 2B). We confirmed the sensitivity of the fatty acid and PL synthesis pathways to environmental changes using a fast nutritional upshift. Addition of glucose and amino acids to a glycerol culture also caused rapid accumulation of PA and PS species that resembled the increases observed following translation inhibition (Fig. S7).

10.1128/mBio.02703-19.8 FIG S7 Response of PL species (counts per total PE volume) to addition of glucose and amino acids at *t* = 0 to a glycerol culture of wild-type E. coli. Values are normalized such that the concentrations before *t* = 0 are averaged to a value of 1. Data points indicate individual measurements from a single time series. Download FIG S7, PDF file, 0.3 MB. Copyright © 2020 Noga et al. 2020 Noga et al. This content is distributed under the terms of the Creative Commons Attribution 4.0 International license.

### Moderate to high concentrations of ppGpp regulate PL synthesis via posttranslational control.

**FIG 4:**
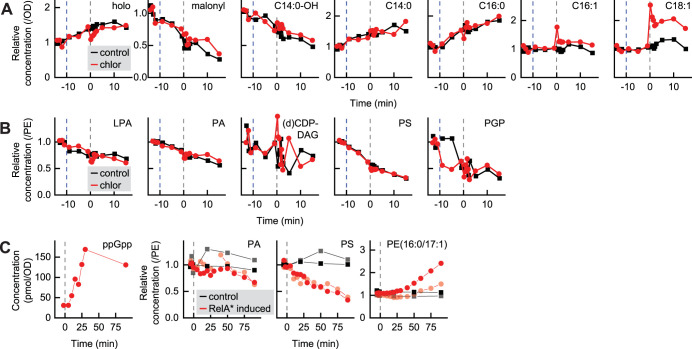
PlsB activity is suppressed by moderate to high concentrations of ppGpp via posttranslational inhibition. (A and B) Responses of ACP intermediate pools (A) and PL intermediate pools (B) to maximal overexpression of ppGpp synthesis enzyme RelA*. RelA* expression was induced by addition of 40 ng/ml doxycycline at −10 min (dashed blue line), followed 10 min later by chloramphenicol addition (dashed gray line at 0 min). (C) Response of concentrations of PL intermediates and cyclopropyl-PE to mild RelA* overexpression, triggered by addition of 1 ng/ml doxycycline (dashed gray line at 0 min). Each time series represents one independent biological replicate.

In order to clearly discern the effects of ppGpp on the fatty acid and PL synthesis pathways without complications introduced by translation inhibition, we monitored the fatty acid and PL synthesis pathways immediately after inducing RelA*. The responses of the fatty acid and PL synthesis pathways were again consistent with PlsB inhibition causing long-chain ACP to accumulate, which depletes malonyl-ACP by inhibiting ACC (Fig. 4A). PL intermediates also responded in a manner consistent with PlsB inhibition: levels of LPA species steadily decreased, followed by PA, PS, and PGP (Fig. 4B). Addition of chloramphenicol 10 min following RelA* induction caused an increase in unsaturated long-chain ACP levels, though ppGpp appears to attenuate the response of the PL pathway to translation inhibition.

We next sought to evaluate whether ppGpp concentrations far below those occurring during the stringent response would also be capable of regulating PlsB via posttranslational control. As the effects of posttranslational control can be distinguished from those of transcriptional regulation by response time, we closely followed the dynamics of the PL pathway after mildly inducing ppGpp synthesis with a low doxycycline concentration. ppGpp concentrations increased within 10 min of RelA* induction and reached the elevated steady-state concentration (∼150 pmol/OD) by 20 min. Concentrations of PS begin to decrease within 15 min (Fig. 4C), consistent with posttranslational inhibition of PL synthesis. To verify that the immediate PS depletion had occurred too quickly to be explained by transcriptional regulation, we compared the PS response kinetics with the kinetics of a ppGpp-driven response known to be mediated by transcriptional control. Cyclopropyl PL is produced from unsaturated fatty acids of membrane PL by the activity of the enzyme Cfa, expression of which is induced via transcriptional control by ppGpp (28). Cyclopropyl PL began to accumulate 25 min after RelA* induction, well after the PS decrease was established. We conclude that ppGpp concentrations well below stringent response concentrations inhibit PlsB via a posttranslational mechanism.

## DISCUSSION

Over several decades, biochemical and genetic research has extensively characterized the pathways and the enzymes that produce the building blocks of bacterial membranes (4, 29, 30). Many control mechanisms acting on both the transcriptional and posttranslational levels have been proposed to maintain membrane homeostasis. For instance, each of the enzymes ACC (31), FabH (32), FabZ (33), FabI (34), LpxC (35), and PlsB (36) have been suggested to contribute to membrane synthesis regulation. However, the existence of a control mechanism does not necessarily indicate that it is used to regulate total membrane synthesis flux during steady-state growth. Furthermore, models derived from biochemical and genetic studies can overlook self-regulating mechanisms that occur automatically in a metabolic pathway, such as the coupling of C14:0-OH-ACP concentrations to PL flux. The primary value of our work is that it reveals the regulatory mechanisms that are actually used by growing E. coli cells to regulate membrane biosynthesis. Both our data and our modeling indicate that only two of the many control mechanisms previously identified are sufficient to regulate total PL biosynthesis during steady-state growth. Specifically, we found that cellular demand for membrane synthesis is communicated to PlsB via an undiscovered posttranslational mechanism. In turn, PlsB activity regulates fatty acid synthesis by consuming long-chain ACP species, relieving feedback inhibition of ACC (20) and increasing fatty acid flux. This demand-level flux regulation is consistent with metabolic control theory (37).

Our measurements of enzyme concentrations indicate that PL flux is regulated primarily via posttranslational control of enzyme activity, rather than via transcriptional regulation. The concentrations of the pathway enzymes with the greatest control over PL flux (ACC and PlsB) are maintained at nearly invariant levels across a 3-fold range of μ. Although it has been long accepted that PlsB activity is adjusted via posttranslational control and not via transcriptional control (4), the strong flux control of ACC (31) and the growth-regulated transcription of the *accBC*, *accA*, and *accD* genes (38) inspired the suggestion that PL flux might be coupled with μ via transcriptional regulation. Instead, we found that transcriptional regulation of fatty acid and PL synthesis genes stabilizes the concentrations of fatty acid and PL synthesis enzymes across μ. The correlations between concentrations of several enzymes (e.g., FabF) and PL flux that we observed are unlikely to increase total PL flux but rather likely regulate aspects of membrane metabolism aside from PL flux, such as membrane fluidity. Maintaining a stable membrane synthesis capacity enables the cell to quickly respond to any change in membrane demand at the cost of expressing enzymes that remain less active at low or moderate μ (39).

Our data are also essential for evaluating proposed models of LPS synthesis regulation. Most importantly, both our model and our experiments indicate that PL flux—and thus, PlsB activity—controls concentrations of LPS precursor C14:0-OH-ACP. This tightly links fluxes into the PL and LPS synthesis pathways, as varying PL synthesis by adjusting either PlsB or ACC activity would naturally vary LPS synthesis in parallel. Surprisingly, we found that flux into the LPS synthesis pathway is not adjusted by variations in LpxC concentrations, as LpxC concentrations remain constant despite a 3-fold change in μ. LPS flux may be varied instead by FabZ, concentrations, which increased by 50% over the 3-fold range of μ sampled (see Fig. S2 in the supplemental material). Our model predicts that this would attenuate the effects of increasing C14:0-OH-ACP concentrations caused by elevated fatty acid synthesis and divert flux away from LPS synthesis without affecting PL flux. LpxC concentrations may be stabilized across μ by degradation by the FtsH protease (40). Degradation of LpxC by FtsH might be used as an emergency brake to rapidly halt LPS synthesis during growth arrest or growth transitions to prevent accumulation of toxic LPS intermediates. Comprehensive characterization and modeling (41) of LPS substrates and enzymes across several steady-state conditions, as we have done for PL synthesis, will be necessary to determine how LPS synthesis is varied.

Although PlsB determines PL flux during steady-state growth, this control is not absolute, as fatty acid and PL flux (and likely LPS flux) are also sensitive to acetyl-CoA concentrations and ACC activity. The sensitivity of membrane synthesis to protein synthesis arrest demonstrates the responsiveness of membrane synthesis to carbon metabolism. While this sensitivity facilitates rapid adaptation of PL flux to environmental changes, the tight connection between protein synthesis, central carbon metabolism, and membrane biogenesis demands additional regulation (including inhibition by ppGpp) to rebalance the pathway with μ and prevent PL overflow. If protein synthesis were inhibited in a manner that does not decrease carbon flow into the fatty acid pathway (e.g., by nitrogen starvation), continued PL and LPS synthesis would outpace synthesis of the lipoproteins that tether the outer membrane to the peptidoglycan layer. Inhibition of PL and LPS pathways by ppGpp would thus prevent production of excess membrane, enforcing the coupling of PL and lipoprotein synthesis. Consistent with this notion, Δ*relA* strains generate higher quantities of extracellular PL and LPS, likely as outer membrane vesicles (42).

Identifying the specific signal that controls PlsB will reveal how membrane synthesis is synchronized with growth. But what controls PlsB? Posttranslational control of PlsB by moderately high concentrations of ppGpp (≥150 pmol/OD) at least partly contributes to steady-state membrane synthesis regulation, but likely only during nutritional downshifts, stress, or very slow steady-state growth (μ < 0.5 h^−1^) when such concentrations are achieved. Whether basal ppGpp concentrations observed during fast growth (μ > 0.5 h^−1^, ppGpp < 60 pmol/OD) also regulates PL synthesis via PlsB control remains unclear. *In vivo* experiments such as ours cannot determine the mechanism by which ppGpp inhibits PlsB. Although it was demonstrated previously that high concentrations of ppGpp directly inhibit PlsB (25), multiple groups were unable to observe ppGpp inhibition of PlsB *in vitro* when acyl-ACP was used as a substrate (43, 44). ppGpp may therefore inhibit PlsB indirectly via a regulator or a cellular process that interacts with PlsB. Immunoprecipitation experiments revealed several proteins that interact with PlsB, including ACP and PssA, as well as some (PlsX and YbgC) whose roles are unclear (45), suggesting that PlsB forms part of a PL synthesis complex that may couple PlsB activity with μ. As PL synthesis flux has been observed to oscillate with the cell division cycle in E. coli and other bacteria (46), PlsB may also be regulated by the divisome or by septum formation. Degradation of PL by the activity of phospholipases may also play an important role in membrane homeostasis during steady-state growth (47), as is known to occur during growth and division in eukaryotes (48). In addition to identifying the allosteric regulators of PlsB, studies that integrate connections between PL catabolism and transport with PL synthesis are needed for a comprehensive understanding of membrane homeostasis.

## MATERIALS AND METHODS

### Culture conditions.

Cultures were grown in 250-ml to 1,000-ml Erlenmeyer flasks filled to 10% of nominal volume with MOPS (morpholinepropanesulfonic acid) minimal medium (49) with 9.5 mM NH_4_Cl or ^15^NH_4_Cl and 0.2% (wt/vol) carbon source (acetate, succinate, malate, glycerol, glucose, U-^13^C-glucose [Cambridge Isotope Laboratories], or glucose supplemented with 0.1% Cas-amino acids). Culture flasks were placed in a Grant Instruments Sub Aqua Pro dual water bath at 37°C and agitated by stirring with a 12-mm-long magnetic stir bar (VWR), coupled to a magnetic stir plate (2mag MIXdrive 1 Eco and MIXcontrol 20) set at 1,200 rpm. Growth was monitored by optical density measurement at 600 nm using an Ultrospec 10 cell density meter (GE Healthcare). Samples for ACP, lipid analysis, and proteomics were collected using cultures without isotopic labeling. Samples for nucleotide phosphate measurements were collected from U-^15^N-labeled cultures, and samples for G3P measurements were collected from U-^13^C-labeled cultures.

### Strains and plasmid pRelA*.

All experiments were performed using Escherichia coli K-12 strain NCM3722 (CGSC catalog no. 12355) and its derivatives. NCM3722 *relA*::*kan* was constructed by P1 phage transduction using strain CF7974 [MG1655 Δ*lac* (*rph*^+^) *relA255*::*kan*] as a donor. Plasmid pRelA* was created by cloning DNA encoding residues 1 to 455 from the E. coli RelA protein into BglBrick plasmid pBbS2k (50) (SC101* origin of replication, P_Tet_ promoter, kanamycin resistance). The fluorescent protein mVenus was fused by restriction-digestion to the C terminus of RelA via a glycine-serine linker.

### Metabolite sampling.

Samples for ACP, proteomics, and lipid analysis were acquired by fast quenching of 1 ml of culture sample into 250 μl of ice-cold 10% trichloroacetic acid. After 10 min incubation at 0°C, cells were pelleted by centrifugation and stored at −80°C until analysis. For analysis of nucleotide phosphates and polar metabolites, samples were acquired by the use of a modified fast vacuum filtration method (51). A 1-ml volume of culture was collected by vacuum on a prewetted 2.5-cm-diameter 0.45-μm-pore-size HV Durapore membrane filter. After rapid collection, the filter was immediately placed upside down in quenching solution. For measurement of the nucleotide phosphates, 1 ml of ice-cold 2 M formic acid was used with 10 μl internal standards (IS) mix as a quenching solution, which was subsequently neutralized by the use of 25 μl of 28% ammonium hydroxide. For G3P measurements, 1 ml of a 50:30:20 (vol/vol/vol) mixture of methanol, acetonitrile, and water with 0.1% formic acid with 10 μl internal standard solution (cooled on dry ice) was used as a quenching solution. After 10 min of incubation, cells were washed from the filter, transferred to a tube, and stored at −80°C until analysis.

### Preparation of internal standards.

Isotopically labeled internal standards (IS) were used to control for sampling and measurement variation. For ACP and proteomics assays, U-^15^N E. coli whole-cell extracts were prepared using a MOPS minimal medium culture with ^15^NH_4_Cl as the sole nitrogen source. At OD of ∼0.5, 10% trichloroacetic acid (TCA) was added at a 1:4 ratio to the culture to facilitate quenching of metabolism. After 10 min of incubation on ice, 10-ml single-use IS aliquots were collected by centrifugation and stored at −80°C until the sample preparation step. For the phospholipid measurement, U-^13^C lipid extract was prepared using a culture grown in minimal MOPS medium with 0.2% U-^13^C glucose as the sole carbon source. At OD of ∼0.5, 10% TCA was added at a 1:4 ratio to the culture and insoluble cell material was collected by centrifugation after 10 min of incubation on ice. Pellets were resuspended in a mixture consisting of 75 μl MeOH, 10 μl 15 mM citric acid–20 mM dipotassium phosphate buffer, and 250 μl of methyl-t-butyl ether per 1 ml of initial culture volume. After vortex mixing and 10 min of sonication, phase separation was induced by addition of 70 μl/ml of 15 mM citric acid/20 mM dipotassium phosphate buffer. After further vortex mixing, sonication and 10 min of incubation at room temperature, the phases were separated by 10 min of centrifugation at 4,000 rpm at room temperature. The upper phase was collected, placed in a glass vial, and stored at −20°C until the sample preparation step.

### Instrumentation.

All LC/MS runs were performed using a Agilent LC/MS system consisting of a binary pump (G1312B), an autosampler (G7167A), a temperature-controlled column compartment (G1316A), and a triple-quadrupole (QQQ) mass spectrometer (G6460C) equipped with a standard electrospray ionization (ESI) source, all operated using MassHunter data acquisition software (version 7.0). A mass spectrometer was operated in dynamic multiple-reaction monitoring (MRM) mode using transitions generated *in silico* by the use of a script written in Python, an RDkit library, and chemical structures of the target compound as the input. Transitions for targeted proteomics assays were developed using Skyline (52) based on protein sequences from the Uniprot database.

### LC/MS quantification of ACP intermediates.

Acyl-ACP levels were measured using a published method (19) with minor modifications. Lysis buffer was prepared by suspending an appropriate number of frozen U-^15^N-labeled E. coli pellets in 10 ml of a mixture consisting of 50 mM potassium phosphate buffer (pH 7.2), 6 M urea, 10 mM N-ethyl-maleimide, 5 mM EDTA, and 1 mM ascorbic acid. A 1-ml volume of lysis buffer was added to each of the preparations of TCA-quenched and pelleted cells, and proteins were isolated by chloroform/methanol precipitation as described previously. Protein pellets were resuspended in 10 μl of digestion buffer (4% 2-octyl-glucoside–25 mM potassium phosphate buffer, pH 7.2) and, after addition of 10 μl of 0.1 mg/ml GluC protease (Promega), incubated overnight at 37°C. After quenching was performed by addition of 5 μl MeOH, samples were centrifuged and 10 μl was injected in an LC/MS system. Separation was performed on a CSH C_18_ column (Waters) (2.1 mm by 50 mm, 1.7-μm pore size) held at 80°C using the following binary gradient: 15% B, 3-min ramp to 25%, 9-min increase to 95%, and 1-min hold at 95% B followed by 3 min of reequilibration under the starting conditions (A, 25 mM formic acid; B, 50 mM formic acid) at a flow rate of 0.6 ml/min.

### LC/MS quantification of phospholipids.

The sample preparation procedure used for the phospholipids consisted of a combination of an methyl tert-butyl ether (MTBE) extraction method (53) and an established LC/MS method (54). Pelleted E. coli cells were resuspended in a mixture containing 150 μl of MeOH, 250 μl of U-^13^C E. coli extract prepared as described above, and 250 μl of MTBE. After vigorous vortex mixing and sonication, 125 μl of 15 mM citric acid–20 mM dipotassium phosphate buffer was added to homogenized pellets. Following further vortex mixing and 10 min of incubation at room temperature, liquid phases were separated by centrifugation for 10 min at 20,000 × *g*. A 500-μl volume of the upper phase was moved to a new tube and dried in a vacuum centrifuge (Labconco). Dried lipid films were resuspended in 10 μl of 65:30:5 (vol/vol/vol) isopropanol/acetonitrile/H_2_O, supplemented with 10 mM acetylacetone. After resuspension, 5 μl H_2_O was added to reduce the organic content of the buffer and 5 μl of the resulting mixture was injected into the LC/MS system. Separation was performed on a CSH C_18_ column (Waters) (2.1 mm by 50 mm, 1.7-μm pore size) at 60°C with a flow rate of 0.6 ml/min using the following binary gradient: 25% B, ramp to 56% B in 6 min followed by a linear increase to 80% B in 6 min, 2-min hold at 100% B, and 3 min reequilibration (A, 0.05% NH4OH–water; B, 0.05% NH_4_OH–80% isopropanol–20% acetonitrile [ACN]).

### LC/MS quantification of nucleotides.

Frozen cell extracts were defrosted by 1 to 2 min of incubation in a 37°C water bath and sonicated for 10 min in a water-ice slurry. After 10 min of centrifugation at 20,000 × *g*, samples were loaded on a 1 ml/30 mg Oasis Wax cartridge (Waters) preconditioned with 1 ml of MeOH and 1 ml 50 mM ammonium acetate buffer (pH 4.5). After washing with 1 ml ammonium acetate buffer was performed, analytes were eluted with 200 μl of 2.8% ammonium hydroxide–MeOH/ACN/H_2_O 50:30:20 (vol/vol/vol). After addition of 10 μl of 5% trehalose and brief vortex mixing, the samples were dried in a vacuum centrifuge (Labconco). Dried trehalose-stabilized extracts were redissolved in 20 μl of MeOH/ACN/H_2_O (50:30:20 [vol/vol/vol]) and moved to an autosampler vial for analysis. Separation was performed on an iHilic column (Hilicon) (2.1 mm by 100 mm, 3.5-μm pore size) or a SeQuant Zic-cHILIC column (Merck) (2.1 mm by 100 mm, 3-μm pore size) at 0.3 ml/min using the following binary gradient: 100% B, ramp to 85% B in 1.5 min followed by 10 min of isocratic hold at 85% B and a linear decrease to 30% B in 3 min with a 2-min hold at 30% B and 8 min reequilibration under the initial conditions (A, 3.75 mM ammonium acetate–1.25 mM acetic acid–2 mM acetylacetone–Milli-Q water, B, 11.25 mM ammonium acetate–3.75 mM acetic acid–2 mM acetylacetone–80% ACN). The injection volume was 2 μl.

### LC/MS quantification of G3P.

Stored metabolite extracts were dried down in a vacuum centrifuge (Labconco), redissolved in 20 μl of MeOH:ACN:H_2_O (50:30:20 [vol/vol/vol]), and moved to an autosampler vial for analysis. Separation was performed on an iHilic column (Hilicon) (2.1 mm by 100 mm, 3.5-μm pore size) at 0.3 ml/min using the following binary gradient: 100% B, ramp to 80% B in 10 min followed by linear decrease to 30% B in 3 min, 2-min hold at 30% B, and 8 min reequilibration. The injection volume was 2 μl.

### LC/MS targeted protein quantification.

Relative concentrations of enzymes were measured by targeted proteomics using a modified version of the ACP assay. Lysis buffer was prepared by suspending an appropriate number of frozen U-^15^N-labeled E. coli pellets in 10 ml of 50 mM potassium phosphate buffer (pH 7.2)–6 M urea. To improve the detection of peptides from PlsB and LpxC, U-^15^N-labeled E. coli NCM3722 strains overexpressing PlsB and LpxC were used as internal standards. LpxC was overexpressed using the corresponding plasmid from the ASKA library (55) (National BioResource Project [NIG, Japan]). A 1-ml volume of lysis buffer was added to each of TCA-quenched and pelleted cells, and proteins were isolated by a chloroform/methanol precipitation as described previously. Protein pellets were resuspended in 10 μl of digestion buffer [4% 2-octyl-glucoside–25 mM Tris buffer (pH 8.1) supplemented with 1 mM CaCl_2_ and 5 mM Tris(2-carboxyethyl)phosphine hydrochloride (TCEP)]. Alkylation of cysteine residues was performed by adding 3 μl of 50 mM iodoacetamide followed by 15 min of incubation in darkness. Digestion was performed by adding 10 μl of 0.2 mg/ml Trypsin Gold (Promega) followed by overnight incubation at 37°C. Samples were centrifuged, and 10 μl of the reaction volume was injected in an LC/MS system. Separation was performed on a CSH C_18_ column (Waters) (2.1 mm by 50 mm, 1.7-μm pore size) held at 40°C using a binary gradient: 2% B, 20 min ramp to 25% B, 4 min increase to 40% B, 0.5 ramp to 80%, and 1-min hold at 80% B before 3 min of reequilibration under the starting conditions (A, 25 mM formic acid; B, 50 mM formic acid) at a flow rate of 0.5 ml/min.

### Data analysis.

All LC-MS data files were processed in versions of Skyline (4.x) using a target list chosen on the basis of an *in silico*-generated transition list. Each target compound had a matching isotopically labeled internal standard (IS). Processed data were exported as target compounds and IS peak areas and processed further using a set of Python scripts. Growth rates were obtained from linear fits to log-transformed growth curves. OD-corrected data were obtained by dividing the signal by the OD_600_ value interpolated from the growth curve at the time of sampling. PE-corrected results were produced by dividing the signal by the sum of all of the signals for all phosphatidyl-ethanolamine species from the same measurement (in the case of the phospholipids) or matching sample (in the case of the other assays). In the nucleotide phosphate and G3P assays, absolute concentrations were estimated based on amounts of internal standards in IS-spike solution by assuming that RR = 1 implies equimolar amounts of target compound and IS at the moment of quenching. Correlations in Table 1 were calculated from log(2)-normalized concentrations and PL fluxes using the Descriptive Statistics function (OriginPro v. 2015).

### Mathematical modeling.

The computational model was constructed and tested using COPASI version 4.24 (56). Full details of the model are described in Text S1 in the supplemental material. Results from sensitivity analysis are included in Fig S8.

10.1128/mBio.02703-19.9 FIG S8 Sensitivity analysis of the fatty acid and phospholipid synthesis model demonstrates that the steady-state metabolite concentration trends simulated from PlsB *V*_max_ variations are robust against variations in model parameters. Parameters were simultaneously adjusted across a 4-fold range centered on the model parameter used. PE flux levels were was adjusted by changing the PlsB *V*_max_ as described in the main text. Download FIG S8, PDF file, 0.3 MB. Copyright © 2020 Noga et al. 2020 Noga et al. This content is distributed under the terms of the Creative Commons Attribution 4.0 International license.

### Data availability.

Mass spectrometry data have been deposited in the EMBL-EBI MetaboLights database (https://academic.oup.com/nar/article/48/D1/D440/5613675; PMID 31691833) with the identifier MTBLS1774. The complete data set can be accessed here: https://www.ebi.ac.uk/metabolights/MTBLS1774. (The authors could not make this MetaboLights record available at the time of this paper’s publication due to circumstances related to the COVID-19 pandemic, but it will be made accessible as soon as possible after publication.)
