# Supplementary figures and images for: Posttranslational Control of PlsB Is Sufficient To Coordinate Membrane Synthesis with Growth in Escherichia coli
Source: mBio. 2020 Aug 18;11(4):e02703-19. doi: 10.1128/mBio.02703-19 (PMC7439487; doi:10.1128/mBio.02703-19)

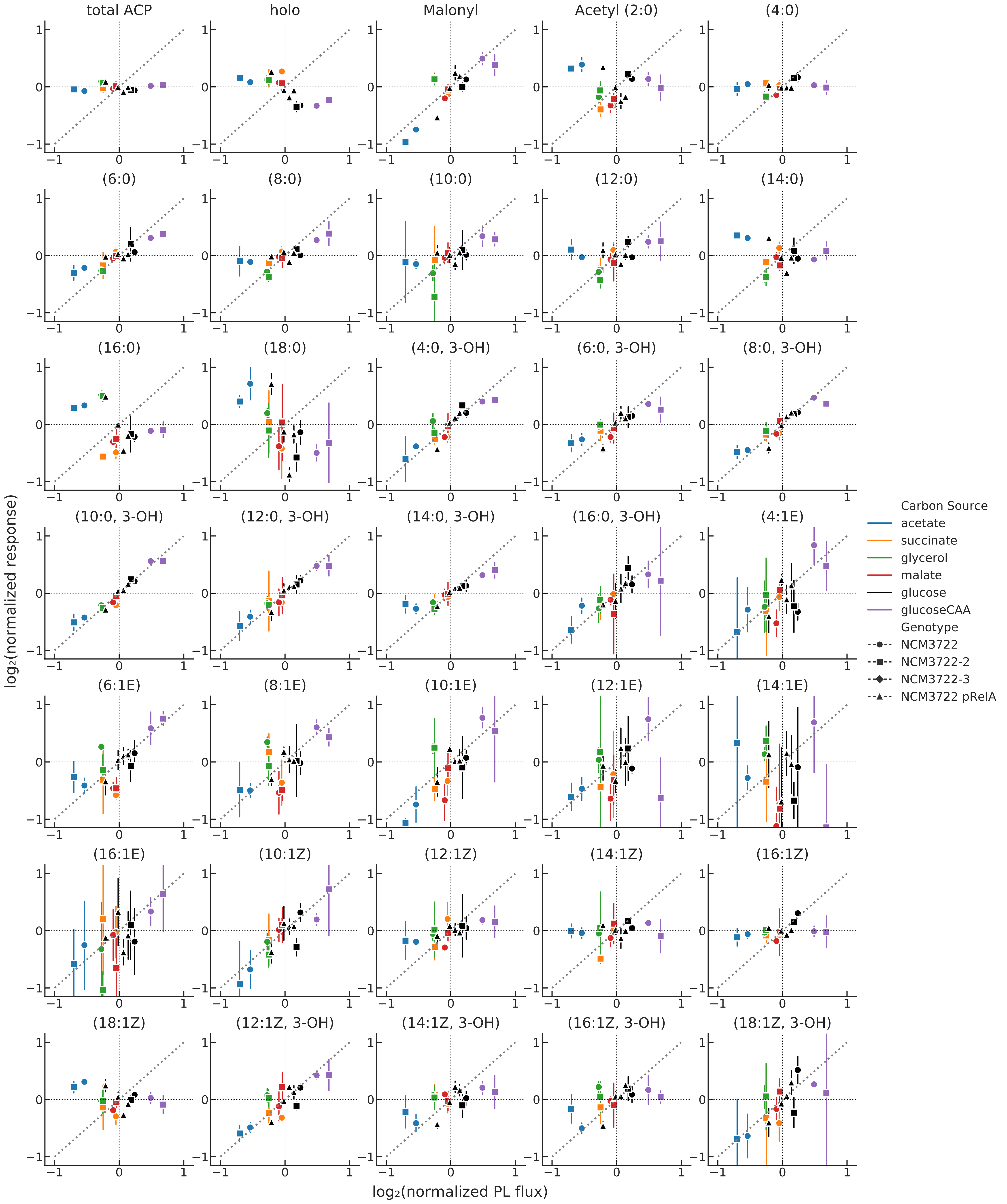

Supplement: FIG S1 [file mBio.02703-19-sf001.pdf]

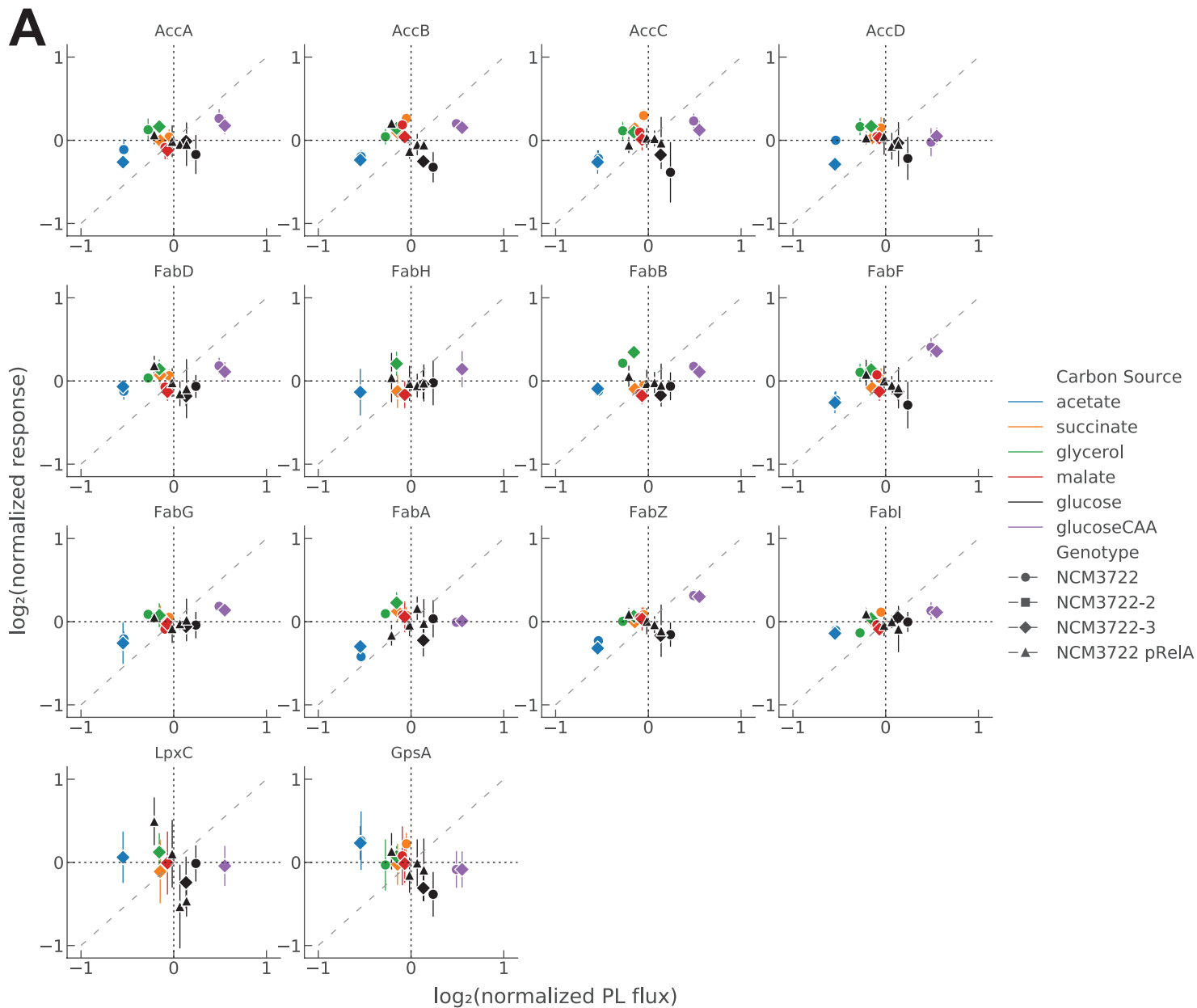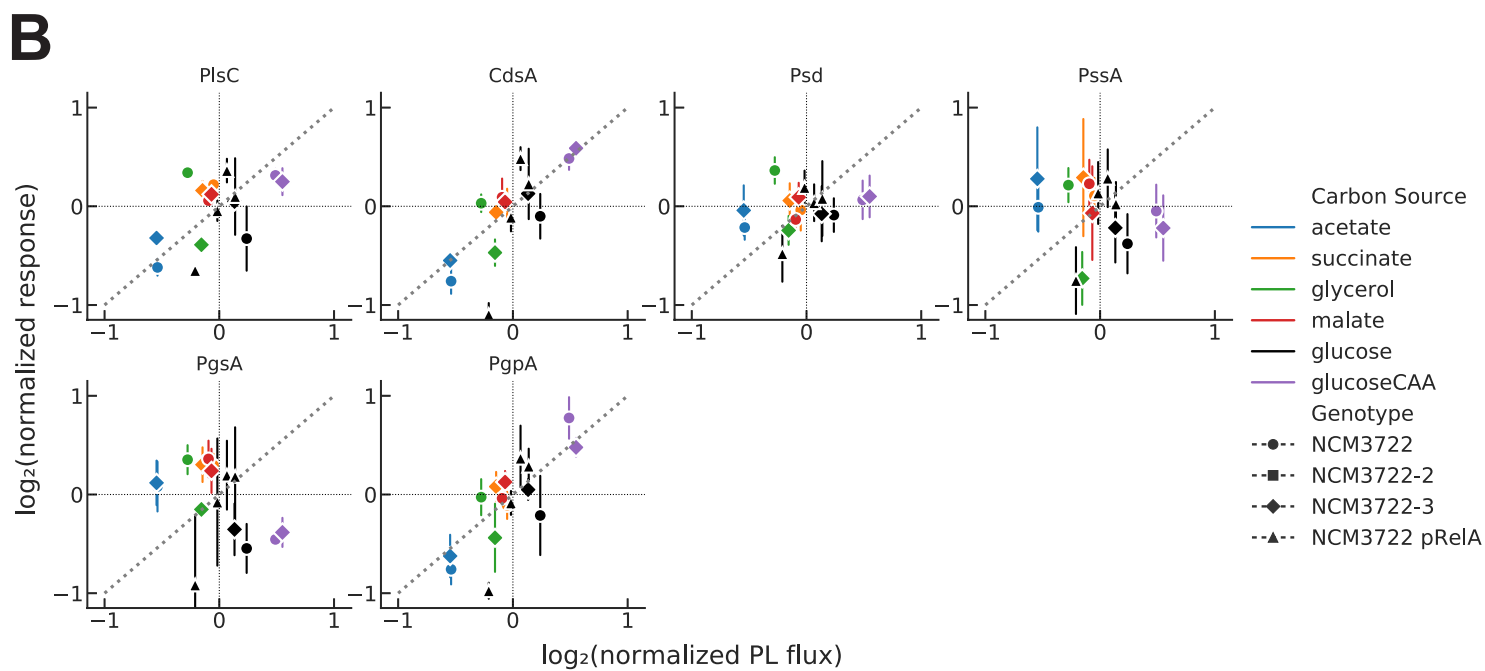

Supplement: FIG S2 [file mBio.02703-19-sf002.pdf]

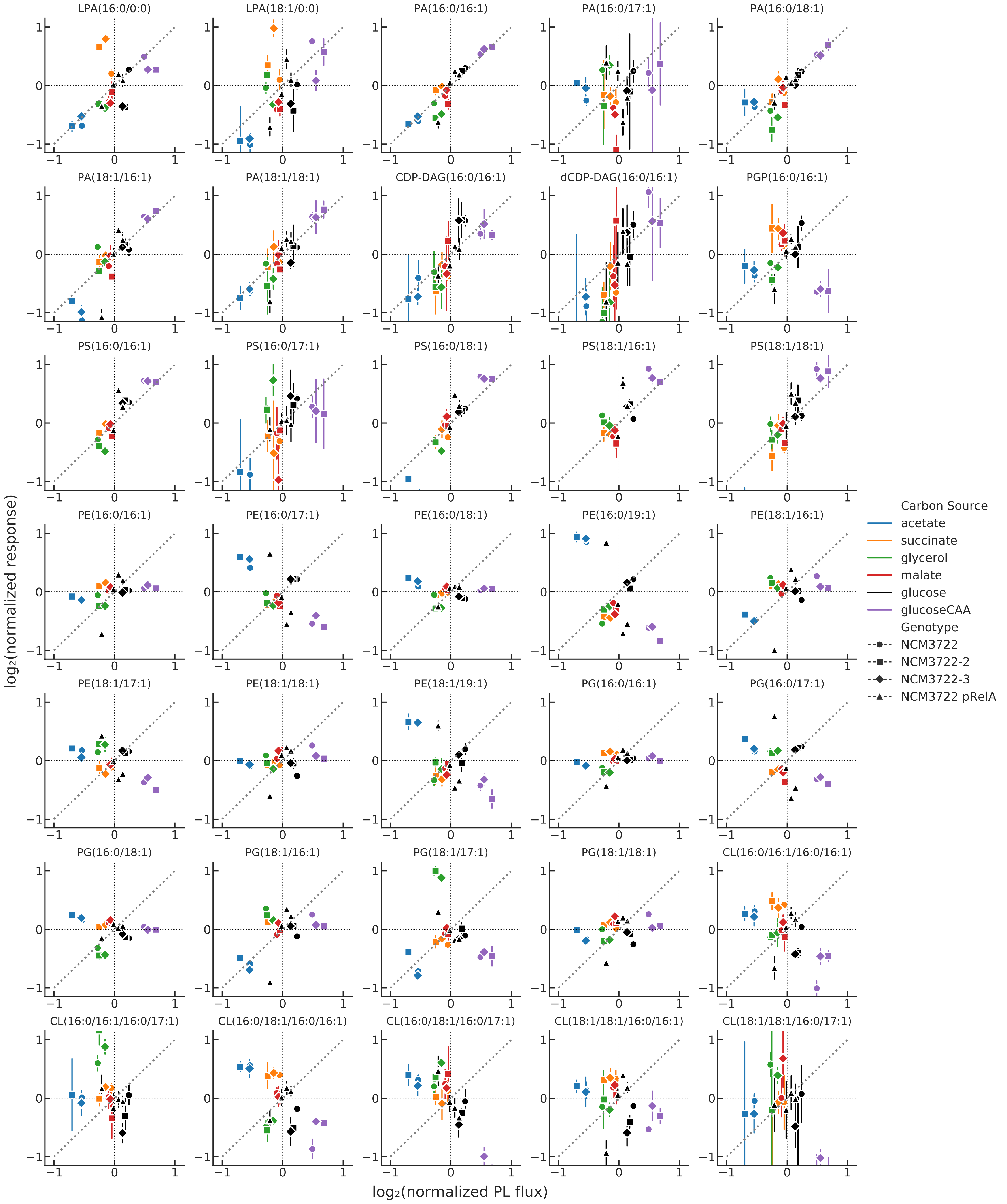

Supplement: FIG S3 [file mBio.02703-19-sf003.pdf]

**A**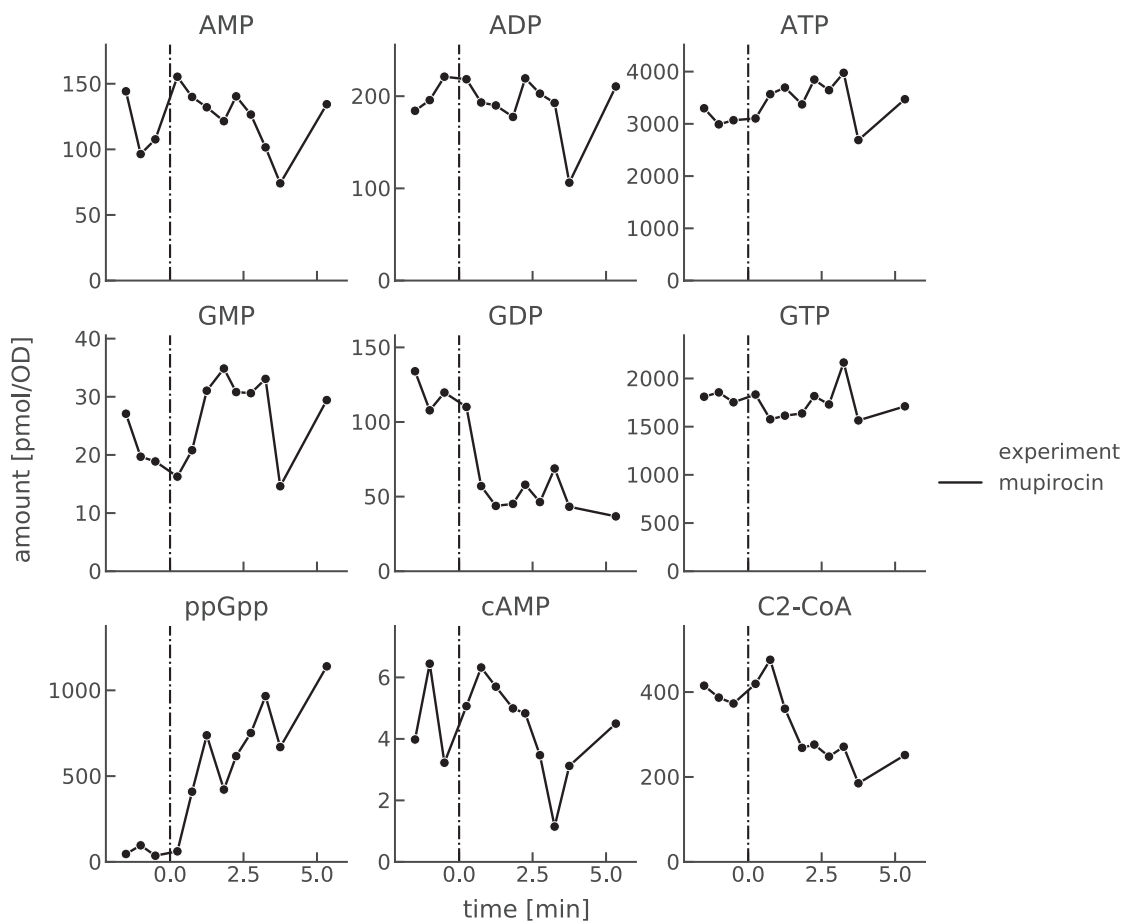**B**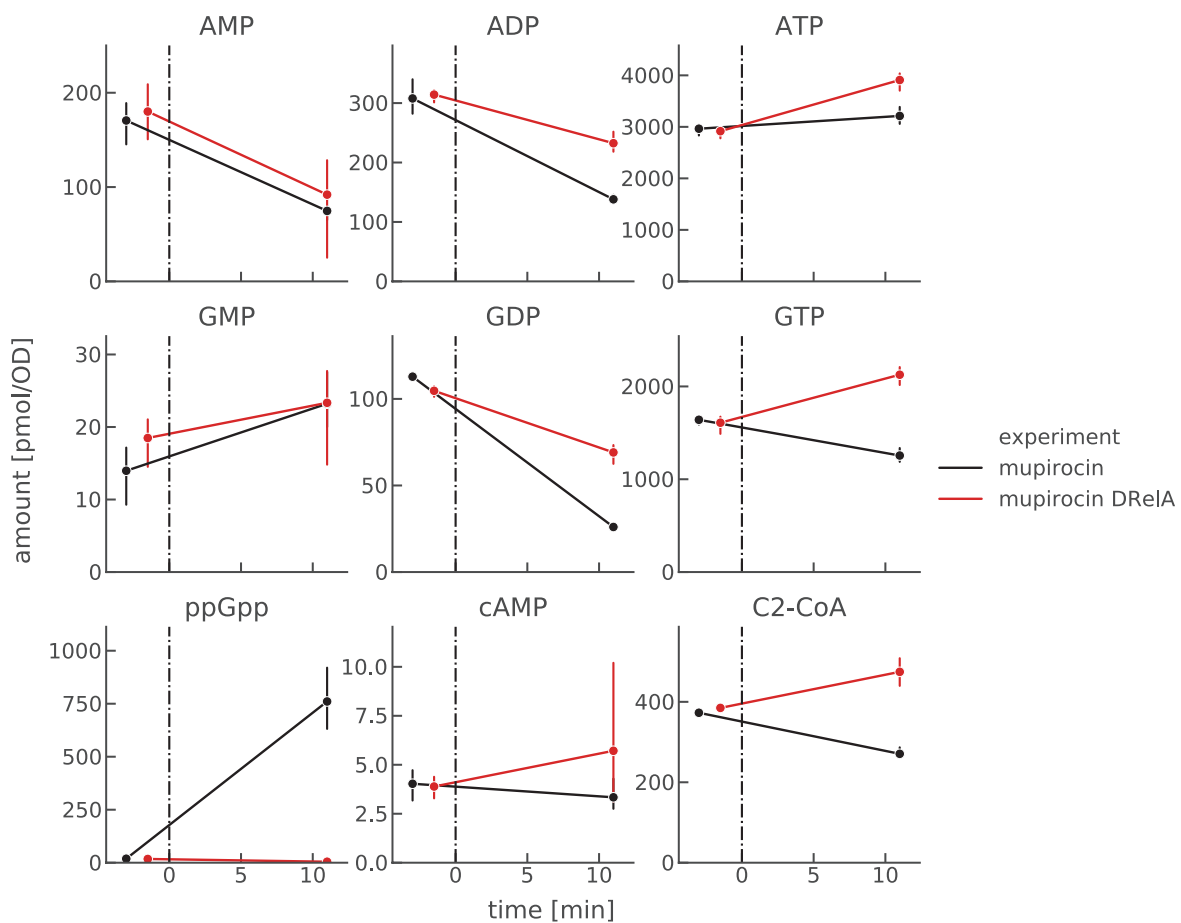

Supplement: FIG S4 [file mBio.02703-19-sf004.pdf]

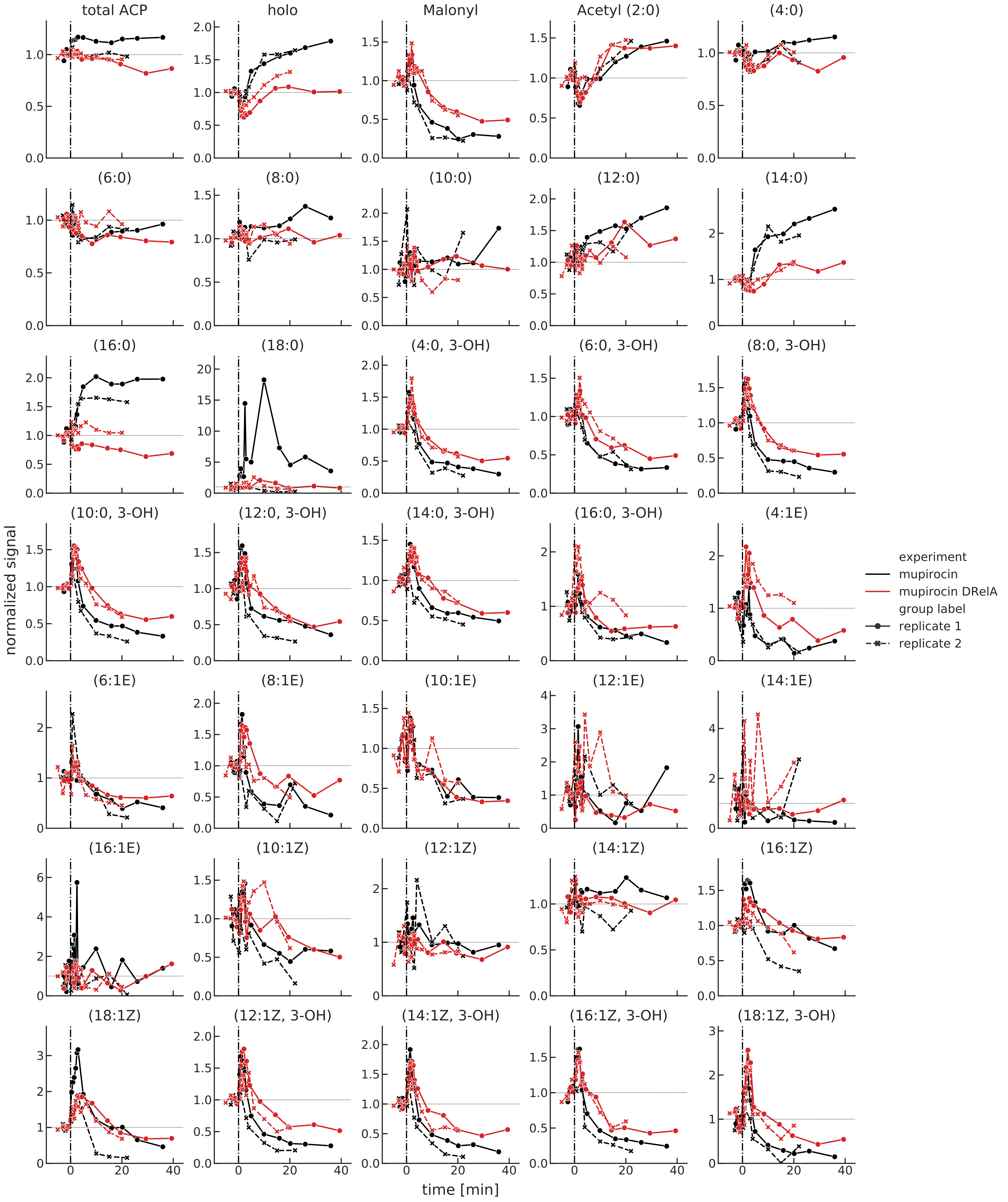

Supplement: FIG S5 [file mBio.02703-19-sf005.pdf]

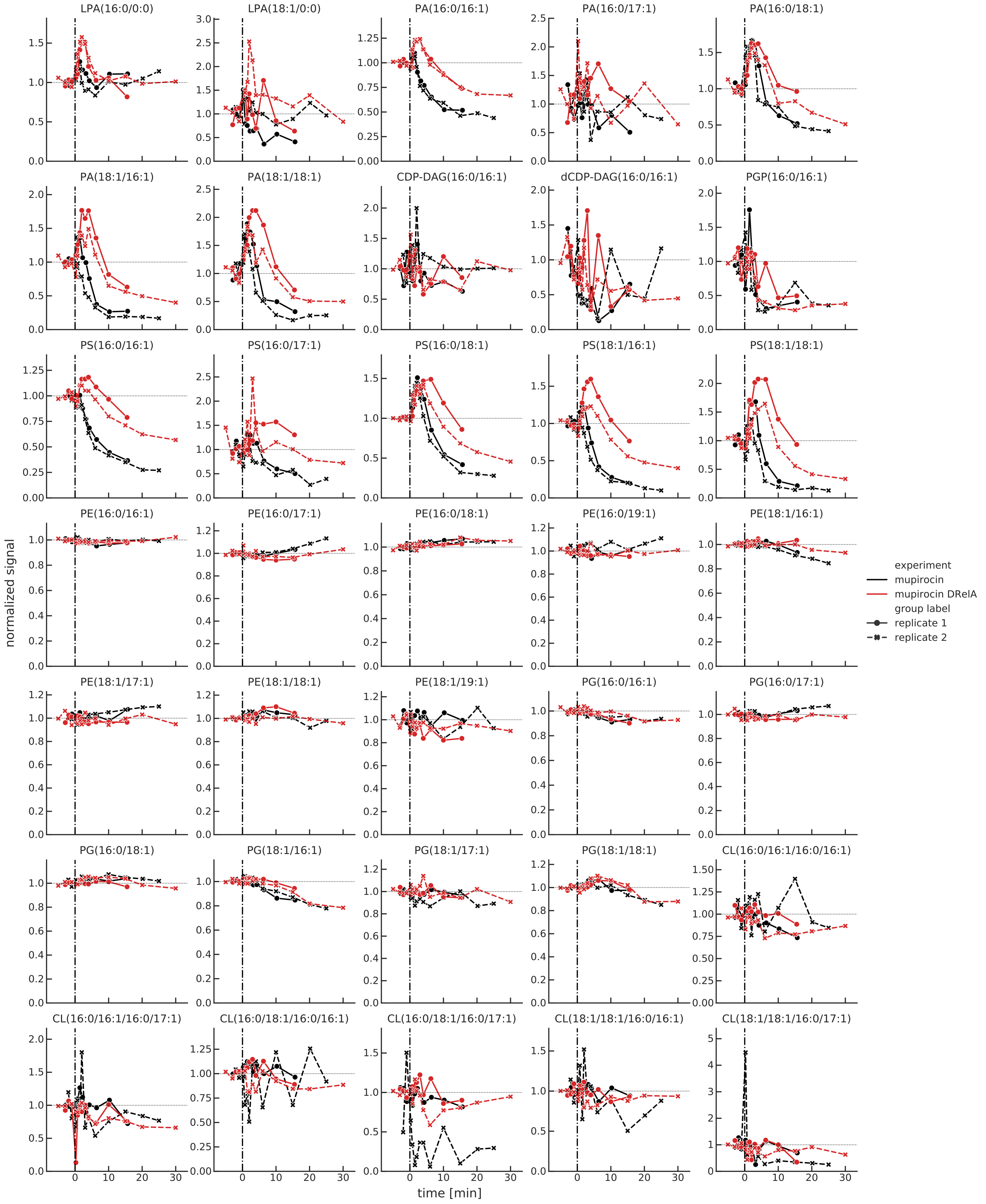

Supplement: FIG S6 [file mBio.02703-19-sf006.pdf]

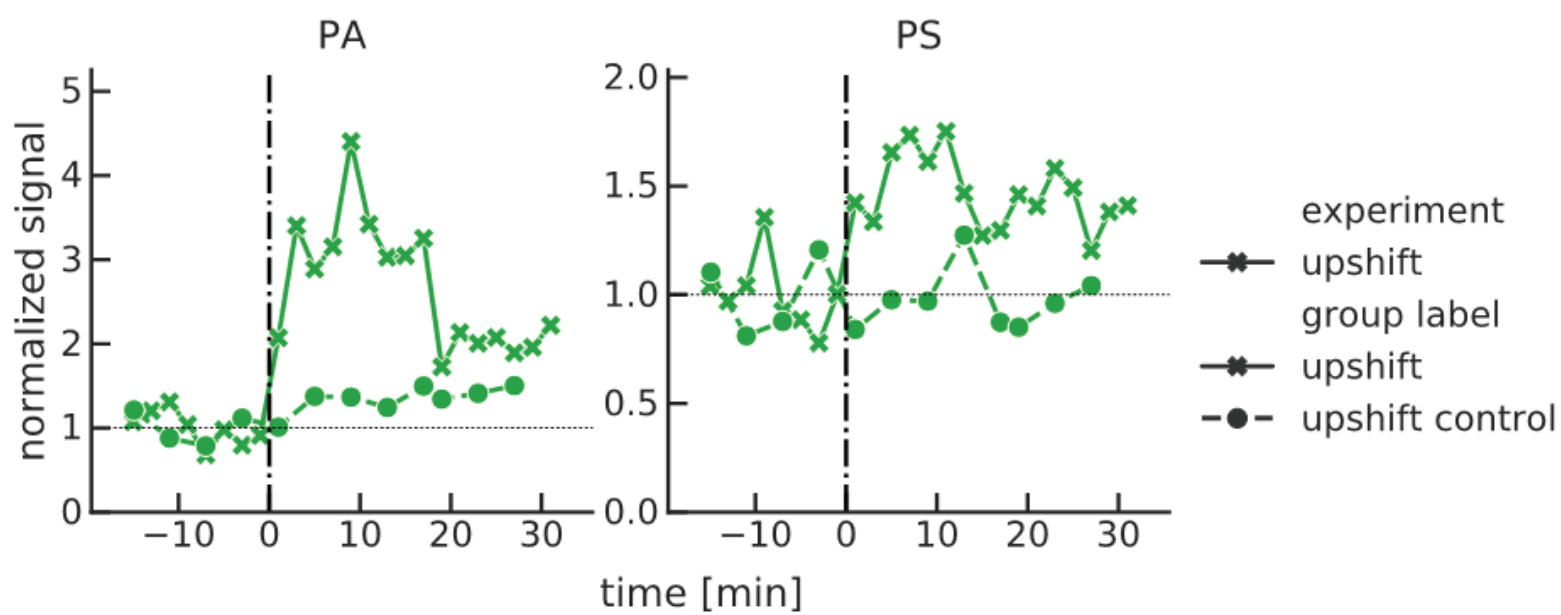

Supplement: FIG S7 [file mBio.02703-19-sf007.pdf]

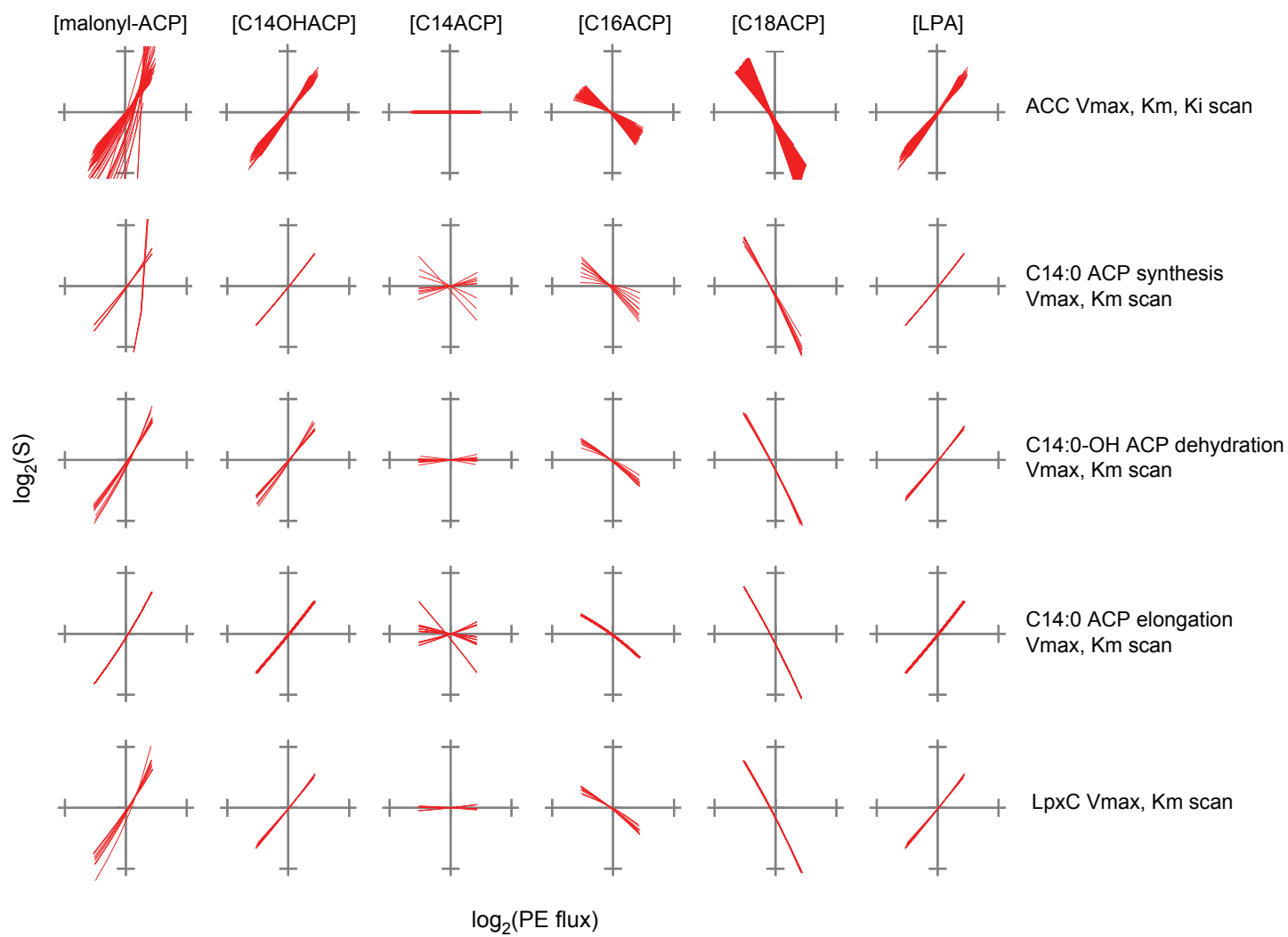

Supplement: FIG S8 [file mBio.02703-19-sf008.pdf]
