# Supplementary material for: Posttranslational Control of PlsB Is Sufficient To Coordinate Membrane Synthesis with Growth in Escherichia coli
Source: mBio. 2020 Aug 18;11(4):e02703-19. doi: 10.1128/mBio.02703-19 (PMC7439487; doi:10.1128/mBio.02703-19)
Supplement: TEXT S1 [file mBio.02703-19-s0001.docx]

**Supplemental Methods**

**Steady-state model description**

The simplified pathway model was set up using COPASI as a series of irreversible Michaelis-Menten equations provided in **Supplemental Table 1**. Most reactions in the fatty acid pathway were excluded in order to determine whether the experimentally-observed trends in intermediate concentrations could be captured in a simplified model. Acetyl-CoA and C16:1-ACP concentrations were fixed at their initial values. For simplicity, V_max_ values and *K*_M_ parameters of each reaction were set to similar values (around 10 and 30 µM, full parameter set given below). For steady-state calculations, we do not claim that the model parameters reflect the exact *in vivo* values. However the values are useful in capturing the basic steady-state behaviours of the system. The simulated steady-state fluxes and metabolite concentrations depicted in **Figure 2** were obtained using the “Parameter Scan” function in COPASI. The differential equations that determine the fluxes through each intermediate pool are defined below.

The strong flux control exerted by PlsB is a consequence of end-product inhibition of ACC by C16:0-ACP and C18:0-ACP, as predicted by metabolic control analysis. Although acyl-ACP species are predicted from experiments to exhibit mixed inhibition of *E. coli* ACC with respect to acetyl-CoA, we use competitive inhibition to minimize the number of parameters in the model. Changes in concentrations of other substrates (e.g. ATP, bicarbonate) or allosteric regulators of ACC (e.g. GlnB) would be expected to exert a similar influence on ACC activity and fatty acid synthesis as variations in ACC V_max_.

| **Values used in mathematical model of fatty acid and PL synthesis pathways.** | | | |
| --- | --- | --- | --- |
| **Initial Species Values** | Species |  | Initial Concentration |
|  | PlsB |  | 1 µmol/l (varied) |
|  | acetyl-CoA |  | 300 µmol/l (fixed) |
|  | malonyl-ACP |  | 0 µmol/l |
|  | C16ACP |  | 0 µmol/l |
|  | C18ACP |  | 0 µmol/l |
|  | LPA |  | 0 µmol/l |
|  | ppGpp |  | 0 µmol/l |
|  | PA |  | 0 µmol/l |
|  | CDPDAG |  | 0 µmol/l |
|  | PS |  | 0 µmol/l |
|  | C161ACP |  | 30 µmol/l (fixed) |
|  | C14BKACP |  | 0 µmol/l |
|  | C14OHACP |  | 0 µmol/l |
|  | C14ACP |  | 0 µmol/l |
|  | C16BKACP |  | 0 µmol/l |
|  | C16OHACP |  | 0 µmol/l |
|  | C18BKACP |  | 0 µmol/l |
|  | C18OHACP |  | 0 µmol/l |
| **Kinetic Parameters** | Reaction | Parameter | Value |
|  | acetyl-CoA carboxylase |  |  |
|  |  | Km | 300 µmol |
|  |  | V | 50 µmol/s |
|  |  | Ki1 | 1 µmol |
|  |  | Ki2 | 1 µmol |
|  | C14 synthesis |  |  |
|  |  | Km | 30 µmol |
|  |  | V | 10 µmol/s |
|  | C16 LPA synthesis |  |  |
|  |  | Km | 30 µmol |
|  |  | kcat | 10 µmol/s |
|  | PA synthesis |  |  |
|  |  | vmax | 20 µmol/s |
|  |  | Kma | 30 µmol |
|  |  | Kmb | 30 µmol |
|  | CDPDAG synthesis |  |  |
|  |  | Km | 30 µmol |
|  |  | V | 10 µmol/s |
|  | PS synthesis |  |  |
|  |  | Km | 30 µmol |
|  |  | V | 10 µmol/s |
|  | PE synthesis |  |  |
|  |  | Km | 30 µmol |
|  |  | V | 10 µmol/s |
|  | C14 reduction |  |  |
|  |  | Km | 30 µmol |
|  |  | V | 10 µmol/s |
|  | LPS initiation |  |  |
|  |  | Km | 30 µmol |
|  |  | V | 10 µmol/s |
|  | C14 dehydration |  |  |
|  |  | Km | 30 µmol |
|  |  | V | 10 µmol/s |
|  | C14 elongation |  |  |
|  |  | vmax | 20 µmol/s |
|  |  | Kma | 30 µmol |
|  |  | Kmb | 30 µmol |
|  | C16 reduction |  |  |
|  |  | Km | 30 µmol |
|  |  | V | 10 µmol/s |
|  | C16 dehydration |  |  |
|  |  | Km | 30 µmol |
|  |  | V | 10 µmol/s |
|  | C16 elongation |  |  |
|  |  | vmax | 20 µmol/s |
|  |  | Kma | 30 µmol |
|  |  | Kmb | 30 µmol |
|  | C18 reduction |  |  |
|  |  | Km | 30 µmol |
|  |  | V | 10 µmol/s |
|  | C18 dehydration |  |  |
|  |  | Km | 30 µmol |
|  |  | V | 10 µmol/s |
|  | C18 LPA synthesis |  |  |
|  |  | Km | 30 µmol |
|  |  | kcat | 10 µmol/s |
