# Supplementary material for: Posttranslational Control of PlsB Is Sufficient To Coordinate Membrane Synthesis with Growth in Escherichia coli
Source: mBio. 2020 Aug 18;11(4):e02703-19. doi: 10.1128/mBio.02703-19 (PMC7439487; doi:10.1128/mBio.02703-19)
Supplement: TABLE S1 [file mBio.02703-19-st001.pdf]

$$\frac{\mathrm{d}([\text{"malonyl-ACP"}] \cdot V_{\mathrm{compartment}})}{\mathrm{d}t} = +V_{\mathrm{compartment}} \cdot \left(\frac{V_{(\text{"acetyl-CoA carboxylase"})} \cdot [\text{"acetyl-CoA"}]}{[\text{"acetyl-CoA"}] + \mathrm{Km}_{(\text{"acetyl-CoA carboxylase"})} \cdot \left(1 + \frac{[\mathrm{C16ACP}]}{\mathrm{Ki1}_{(\text{"acetyl-CoA carboxylase"})}} + \frac{[\mathrm{C18ACP}]}{\mathrm{Ki2}_{(\text{"acetyl-CoA carboxylase"})}}\right)}\right)$$

$$-V_{\mathrm{compartment}} \cdot \left(\frac{V_{(\text{"C14 synthesis"})} \cdot [\text{"malonyl-ACP"}]}{\mathrm{Km}_{(\text{"C14 synthesis"})} + [\text{"malonyl-ACP"}]}\right)$$

$$-V_{\mathrm{compartment}} \cdot$$

$$\left(\frac{\mathrm{vmax}_{(\text{"C14 elongation"})} \cdot [\mathrm{C14ACP}] \cdot [\text{"malonyl-ACP"}]}{\mathrm{Kma}_{(\text{"C14 elongation"})} \cdot \mathrm{Kmb}_{(\text{"C14 elongation"})} + [\mathrm{C14ACP}] \cdot \mathrm{Kmb}_{(\text{"C14 elongation"})} + [\text{"malonyl-ACP"}] \cdot \mathrm{Kma}_{(\text{"C14 elongation"})} + [\mathrm{C14ACP}] \cdot [\text{"malonyl-ACP"}]}\right)$$

$$-V_{\mathrm{compartment}} \cdot$$

$$\left(\frac{\mathrm{vmax}_{(\text{"C16 elongation"})} \cdot [\mathrm{C16ACP}] \cdot [\text{"malonyl-ACP"}]}{\mathrm{Kma}_{(\text{"C16 elongation"})} \cdot \mathrm{Kmb}_{(\text{"C16 elongation"})} + [\mathrm{C16ACP}] \cdot \mathrm{Kmb}_{(\text{"C16 elongation"})} + [\text{"malonyl-ACP"}] \cdot \mathrm{Kma}_{(\text{"C16 elongation"})} + [\mathrm{C16ACP}] \cdot [\text{"malonyl-ACP"}]}\right)$$

$$\frac{\mathrm{d}([\mathrm{C16ACP}] \cdot V_{\mathrm{compartment}})}{\mathrm{d}t} = +V_{\mathrm{compartment}} \cdot \left(\frac{V_{(\text{"C16 dehydration"})} \cdot [\mathrm{C16OHACP}]}{\mathrm{Km}_{(\text{"C16 dehydration"})} + [\mathrm{C16OHACP}]}\right)$$

$$-V_{\mathrm{compartment}} \cdot$$

$$\left(\frac{\mathrm{vmax}_{(\text{"C16 elongation"})} \cdot [\mathrm{C16ACP}] \cdot [\text{"malonyl-ACP"}]}{\mathrm{Kma}_{(\text{"C16 elongation"})} \cdot \mathrm{Kmb}_{(\text{"C16 elongation"})} + [\mathrm{C16ACP}] \cdot \mathrm{Kmb}_{(\text{"C16 elongation"})} + [\text{"malonyl-ACP"}] \cdot \mathrm{Kma}_{(\text{"C16 elongation"})} + [\mathrm{C16ACP}] \cdot [\text{"malonyl-ACP"}]}\right)$$

$$-V_{\mathrm{compartment}} \cdot \left(\frac{\mathrm{kcat}_{(\text{"C16 LPA synthesis"})} \cdot [\mathrm{PlsB}] \cdot [\mathrm{C16ACP}]}{\mathrm{Km}_{(\text{"C16 LPA synthesis"})} + [\mathrm{C16ACP}]}\right)$$

$$\frac{\mathrm{d}([\mathrm{LPA}] \cdot V_{\mathrm{compartment}})}{\mathrm{d}t} = +V_{\mathrm{compartment}} \cdot \left(\frac{\mathrm{kcat}_{(\text{"C18 LPA synthesis"})} \cdot [\mathrm{PlsB}] \cdot [\mathrm{C18ACP}]}{\mathrm{Km}_{(\text{"C18 LPA synthesis"})} + [\mathrm{C18ACP}]}\right)$$

$$+V_{\mathrm{compartment}} \cdot \left(\frac{\mathrm{kcat}_{(\text{"C16 LPA synthesis"})} \cdot [\mathrm{PlsB}] \cdot [\mathrm{C16ACP}]}{\mathrm{Km}_{(\text{"C16 LPA synthesis"})} + [\mathrm{C16ACP}]}\right)$$

$$-V_{\mathrm{compartment}} \cdot \left(\frac{\mathrm{vmax}_{(\text{"PA synthesis"})} \cdot [\mathrm{LPA}] \cdot [\mathrm{C161ACP}]}{\mathrm{Kma}_{(\text{"PA synthesis"})} \cdot \mathrm{Kmb}_{(\text{"PA synthesis"})} + [\mathrm{LPA}] \cdot \mathrm{Kmb}_{(\text{"PA synthesis"})} + [\mathrm{C161ACP}] \cdot \mathrm{Kma}_{(\text{"PA synthesis"})} + [\mathrm{LPA}] \cdot [\mathrm{C161ACP}]}\right)$$

$$\frac{\mathrm{d}([\mathrm{PA}] \cdot V_{\mathrm{compartment}})}{\mathrm{d}t} = +V_{\mathrm{compartment}} \cdot \left(\frac{\mathrm{vmax}_{(\text{"PA synthesis"})} \cdot [\mathrm{LPA}] \cdot [\mathrm{C161ACP}]}{\mathrm{Kma}_{(\text{"PA synthesis"})} \cdot \mathrm{Kmb}_{(\text{"PA synthesis"})} + [\mathrm{LPA}] \cdot \mathrm{Kmb}_{(\text{"PA synthesis"})} + [\mathrm{C161ACP}] \cdot \mathrm{Kma}_{(\text{"PA synthesis"})} + [\mathrm{LPA}] \cdot [\mathrm{C161ACP}]}\right)$$

$$-V_{\mathrm{compartment}} \cdot \left(\frac{V_{(\text{"CDPDAG synthesis"})} \cdot [\mathrm{PA}]}{\mathrm{Km}_{(\text{"CDPDAG synthesis"})} + [\mathrm{PA}]}\right)$$

$$\frac{\mathrm{d}([\mathrm{CDPDAG}] \cdot V_{\mathrm{compartment}})}{\mathrm{d}t} = +V_{\mathrm{compartment}} \cdot \left(\frac{V_{(\text{"CDPDAG synthesis"})} \cdot [\mathrm{PA}]}{\mathrm{Km}_{(\text{"CDPDAG synthesis"})} + [\mathrm{PA}]}\right)$$

$$-V_{\mathrm{compartment}} \cdot \left(\frac{V_{(\text{"PS synthesis"})} \cdot [\mathrm{CDPDAG}]}{\mathrm{Km}_{(\text{"PS synthesis"})} + [\mathrm{CDPDAG}]}\right)$$

$$\frac{\mathrm{d}([\mathrm{PS}] \cdot V_{\mathrm{compartment}})}{\mathrm{d}t} = +V_{\mathrm{compartment}} \cdot \left(\frac{V_{(\text{"PS synthesis"})} \cdot [\mathrm{CDPDAG}]}{\mathrm{Km}_{(\text{"PS synthesis"})} + [\mathrm{CDPDAG}]}\right) - V_{\mathrm{compartment}} \cdot \left(\frac{V_{(\text{"PE synthesis"})} \cdot [\mathrm{PS}]}{\mathrm{Km}_{(\text{"PE synthesis"})} + [\mathrm{PS}]}\right)$$

$$\frac{\mathrm{d}([\mathrm{C14BKACP}] \cdot V_{\mathrm{compartment}})}{\mathrm{d}t} = +V_{\mathrm{compartment}} \cdot \left(\frac{V_{(\text{"C14 synthesis"})} \cdot [\text{"malonyl−ACP"}]}{\mathrm{Km}_{(\text{"C14 synthesis"})} + [\text{"malonyl−ACP"}]}\right) - V_{\mathrm{compartment}} \cdot \left(\frac{V_{(\text{"C14 reduction"})} \cdot [\mathrm{C14BKACP}]}{\mathrm{Km}_{(\text{"C14 reduction"})} + [\mathrm{C14BKACP}]}\right)$$

$$\frac{\mathrm{d}([\mathrm{C14OHACP}] \cdot V_{\mathrm{compartment}})}{\mathrm{d}t} = +V_{\mathrm{compartment}} \cdot \left(\frac{V_{(\text{"C14 reduction"})} \cdot [\mathrm{C14BKACP}]}{\mathrm{Km}_{(\text{"C14 reduction"})} + [\mathrm{C14BKACP}]}\right) - V_{\mathrm{compartment}} \cdot \left(\frac{V_{(\text{"LPS initiation"})} \cdot [\mathrm{C14OHACP}]}{\mathrm{Km}_{(\text{"LPS initiation"})} + [\mathrm{C14OHACP}]}\right) - V_{\mathrm{compartment}} \cdot \left(\frac{V_{(\text{"C14 dehydration"})} \cdot [\mathrm{C14OHACP}]}{\mathrm{Km}_{(\text{"C14 dehydration"})} + [\mathrm{C14OHACP}]}\right)$$

$$\frac{\mathrm{d}([\mathrm{C14ACP}] \cdot V_{\mathrm{compartment}})}{\mathrm{d}t} = -V_{\mathrm{compartment}} \cdot \left(\frac{\mathrm{vmax}_{(\text{"C14 elongation"})} \cdot [\mathrm{C14ACP}] \cdot [\text{"malonyl−ACP"}]}{\mathrm{Kma}_{(\text{"C14 elongation"})} \cdot \mathrm{Kmb}_{(\text{"C14 elongation"})} + [\mathrm{C14ACP}] \cdot \mathrm{Kmb}_{(\text{"C14 elongation"})} + [\text{"malonyl−ACP"}] \cdot \mathrm{Kma}_{(\text{"C14 elongation"})} + [\mathrm{C14ACP}] \cdot [\text{"malonyl−ACP"}]}\right) + V_{\mathrm{compartment}} \cdot \left(\frac{V_{(\text{"C14 dehydration"})} \cdot [\mathrm{C14OHACP}]}{\mathrm{Km}_{(\text{"C14 dehydration"})} + [\mathrm{C14OHACP}]}\right)$$

$$\frac{\mathrm{d}([\mathrm{C16BKACP}] \cdot V_{\mathrm{compartment}})}{\mathrm{d}t} = +V_{\mathrm{compartment}} \cdot \left(\frac{\mathrm{vmax}_{(\text{"C14 elongation"})} \cdot [\mathrm{C14ACP}] \cdot [\text{"malonyl−ACP"}]}{\mathrm{Kma}_{(\text{"C14 elongation"})} \cdot \mathrm{Kmb}_{(\text{"C14 elongation"})} + [\mathrm{C14ACP}] \cdot \mathrm{Kmb}_{(\text{"C14 elongation"})} + [\text{"malonyl−ACP"}] \cdot \mathrm{Kma}_{(\text{"C14 elongation"})} + [\mathrm{C14ACP}] \cdot [\text{"malonyl−ACP"}]}\right) - V_{\mathrm{compartment}} \cdot \left(\frac{V_{(\text{"C16 reduction"})} \cdot [\mathrm{C16BKACP}]}{\mathrm{Km}_{(\text{"C16 reduction"})} + [\mathrm{C16BKACP}]}\right)$$

$$\frac{\mathrm{d}([\mathrm{C16OHACP}] \cdot V_{\mathrm{compartment}})}{\mathrm{d}t} = +V_{\mathrm{compartment}} \cdot \left(\frac{V_{(\text{"C16 reduction"})} \cdot [\mathrm{C16BKACP}]}{\mathrm{Km}_{(\text{"C16 reduction"})} + [\mathrm{C16BKACP}]}\right) - V_{\mathrm{compartment}} \cdot \left(\frac{V_{(\text{"C16 dehydration"})} \cdot [\mathrm{C16OHACP}]}{\mathrm{Km}_{(\text{"C16 dehydration"})} + [\mathrm{C16OHACP}]}\right)$$

$$\frac{\mathrm{d}([\mathrm{C18BKACP}] \cdot V_{\mathrm{compartment}})}{\mathrm{d}t} = +V_{\mathrm{compartment}} \cdot \left(\frac{\mathrm{vmax}_{(\text{"C16 elongation"})} \cdot [\mathrm{C16ACP}] \cdot [\text{"malonyl−ACP"}]}{\mathrm{Kma}_{(\text{"C16 elongation"})} \cdot \mathrm{Kmb}_{(\text{"C16 elongation"})} + [\mathrm{C16ACP}] \cdot \mathrm{Kmb}_{(\text{"C16 elongation"})} + [\text{"malonyl−ACP"}] \cdot \mathrm{Kma}_{(\text{"C16 elongation"})} + [\mathrm{C16ACP}] \cdot [\text{"malonyl−ACP"}]}\right) - V_{\mathrm{compartment}} \cdot \left(\frac{V_{(\text{"C18 reduction"})} \cdot [\mathrm{C18BKACP}]}{\mathrm{Km}_{(\text{"C18 reduction"})} + [\mathrm{C18BKACP}]}\right)$$

$$\frac{\mathrm{d}([\mathrm{C18OHACP}] \cdot V_{\mathrm{compartment}})}{\mathrm{d}t} = +V_{\mathrm{compartment}} \cdot \left(\frac{V_{(\text{"C18 reduction"})} \cdot [\mathrm{C18BKACP}]}{\mathrm{Km}_{(\text{"C18 reduction"})} + [\mathrm{C18BKACP}]}\right) - V_{\mathrm{compartment}} \cdot \left(\frac{V_{(\text{"C18 dehydration"})} \cdot [\mathrm{C18OHACP}]}{\mathrm{Km}_{(\text{"C18 dehydration"})} + [\mathrm{C18OHACP}]}\right)$$

$$\frac{\mathrm{d}([\mathrm{C18ACP}] \cdot V_{\mathrm{compartment}})}{\mathrm{d}t} = +V_{\mathrm{compartment}} \cdot \left(\frac{V_{(\text{"C18 dehydration"})} \cdot [\mathrm{C18OHACP}]}{\mathrm{Km}_{(\text{"C18 dehydration"})} + [\mathrm{C18OHACP}]}\right) - V_{\mathrm{compartment}} \cdot \left(\frac{\mathrm{kcat}_{(\text{"C18 LPA synthesis"})} \cdot [\mathrm{PlsB}] \cdot [\mathrm{C18ACP}]}{\mathrm{Km}_{(\text{"C18 LPA synthesis"})} + [\mathrm{C18ACP}]}\right)$$
